# Structural basis for the synergistic assembly of the snRNA export complex

**DOI:** 10.1038/s41594-025-01595-5

**Published:** 2025-07-03

**Authors:** Etienne Dubiez, William Garland, Maja Finderup Brask, Elisabetta Boeri Erba, Torben Heick Jensen, Jan Kadlec, Stephen Cusack

**Affiliations:** 1 Université Grenoble Alpes, CNRS, CEA, IBS, Grenoble, France; 2 European Molecular Biology Laboratory, Grenoble, France; 3 Department of Molecular Biology and Genetics, Aarhus University, Aarhus, Denmark

**Keywords:** Electron microscopy, RNA transport

## Abstract

The nuclear cap-binding complex (CBC) and its partner Arsenite-Resistance Protein 2 (ARS2) regulate the fate of RNA polymerase II transcripts via mutually exclusive interactions with RNA effectors. One such effector is PHAX, which mediates the nuclear export of U-rich small nuclear RNAs (snRNAs). Here we present the cryo-electron microscopy structure of the human snRNA export complex comprising phosphorylated PHAX, CBC, CRM1–RanGTP and capped RNA. The central region of PHAX bridges CBC to the export factor CRM1–RanGTP, while also reinforcing cap dinucleotide binding. Additionally, PHAX interacts with a distant region of CRM1, facilitating contacts of the essential phosphorylated region of PHAX with the prominent basic surface of RanGTP. CBC engagement within the snRNA export complex is incompatible with its binding to other RNA effectors such as ALYREF or NCBP3. We demonstrate that snRNA export complex formation requires synergistic binding of all its components, which in turn displaces ARS2 from CBC and commits the complex for export.

## Abstract (web summary)

Dubiez and colleagues present a cryo-EM structure of the complex responsible for nuclear export of pre-small nuclear RNAs, comprising CBC–PHAX–CRM1–RanGTP. The structure provides insights into the complex architecture, assembly and target RNA recognition.

## Main

Eukaryotic transcription generates vast amounts of RNA^1,2^. While useful transcripts are processed and mainly exported to the cytoplasm, the majority of RNAs are degraded in the nucleus^3^. How transcripts are sorted into different fate pathways is a central question underlying nuclear RNA metabolism.

During early RNA polymerase II (Pol II) transcription, nascent transcripts acquire an m^7^G-cap at their 5′ end, which is rapidly bound by the nuclear cap-binding complex (CBC) composed of CBP80 (also known as NCBP1) and CBP20 (NCBP2)^4,5^. While the cap is recognized by CBP20, CBP80 interacts with numerous factors^5–10^. An important partner of CBC is the Arsenite-Resistance Protein 2 (ARS2), whose C-terminus binds in a groove formed between CBP20 and CBP80 (ref. ^9^). The CBC–ARS2 (CBCA) complex is a key regulator influencing transcriptional and post-transcriptional fate decisions^11^. It is thought that whether transcripts undergo processing, export or degradation depends on dynamic CBCA interactions with competing, mutually exclusive RNA effectors^7,11,12^. These effectors, including the Phosphorylated Adaptor for RNA Export (PHAX), NCBP3 and ZC3H18 (a component of the nuclear exosome targeting (NEXT) complex), bind ARS2 via their ARS2 recognition motifs (ARMs) and the CBC through tryptophan-containing helices^7,13–15^. How these interactions orchestrate the competitive interplay of RNA effectors remains unclear.

**Fig. 1:**
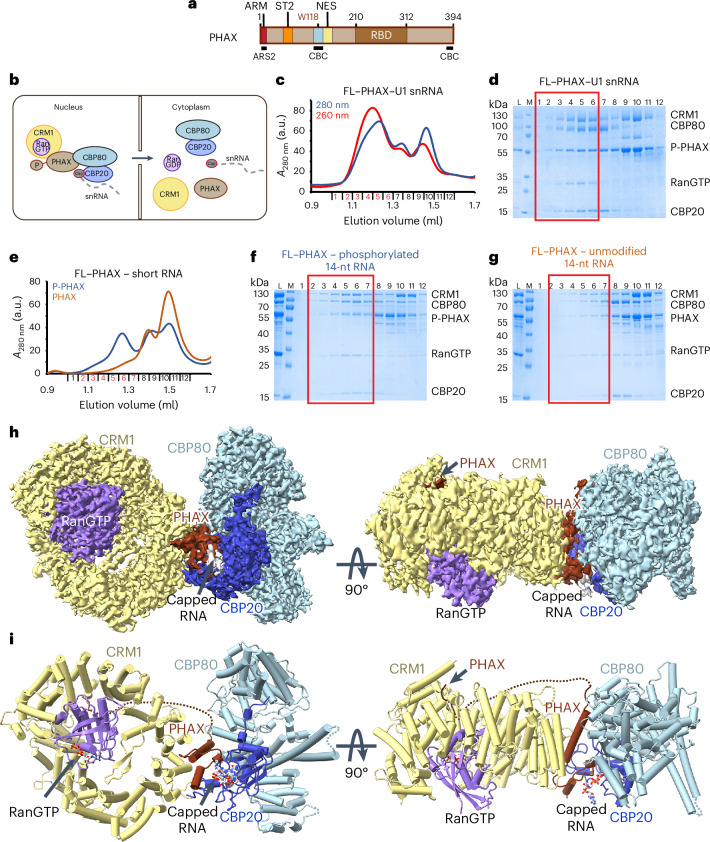
Structural characterization of the snRNA export complex. **a**, Schematic representation of the PHAX domain structure. The positions of the ARS2 (ARM) and CBC-binding regions (W118) are shown. **b**, A schematic model of snRNA export mediated by the snRNA export complex assembled in the nucleus and disassembled in the cytoplasm. **c**,**d**, The Superdex 200 gel filtration elution profile (**c**) and SDS-PAGE analysis (**d**) of fractions 1–12 of the snRNA complex reconstituted with capped U1 snRNA. The five proteins co-elute in the first peak highlighted with the red rectangle in **d**. L, input sample loaded onto the column; M, Mw marker. **e**, Overlay of Superdex 200 gel filtration elution profiles of the snRNA complex reconstitutions with a short capped 14-nt RNA using phosphorylated (P-PHAX) or unmodified PHAX. The complex elutes in fractions highlighted in red. **f**,**g**, SDS-PAGE analysis of fractions 1–12 of the gel filtration elution profiles shown in **e** using P-PHAX (**f**) and unmodified PHAX (**g**). The red rectangle shows fractions containing the snRNA export complex. In **g**, the complex formation is less efficient and CBC co-elutes mostly with PHAX in fractions 8 and 9. **h**, The cryo-EM map used to build the snRNA export complex structure containing a 14-nt capped RNA. The map is colored according to the molecules location in the structure: CBP80 is in light blue, CBP20 in blue, capped RNA in gray, PHAX in dark brick, CRM1 in yellow and Ran in violet. **i**, Ribbon representation of the snRNA export complex. RNA and GTP are shown as sticks. Source data

Correct RNA sorting is particularly crucial for short Pol II transcripts, as functionally important RNAs (for example, small nuclear RNAs (snRNAs)) must be positively selected amidst a vast background of nonproductive RNAs destined for decay^12^. Precursor (pre-)spliceosomal U rich snRNAs, U1, U11, U2, U12, U4 and U5 are transcribed by Pol II^16^. Following 5′ end capping and 3′ end processing by the Integrator complex, snRNAs temporarily transit through Cajal bodies, where a specific snRNA nuclear export complex is assembled^17^. A key element of this complex is PHAX (Fig. 1a), which connects the CBC-bound transcript with the nuclear export factor CRM1 (XPO1), itself bound to the small GTPase Ran and GTP (RanGTP)^18^. PHAX recruits CRM1–RanGTP via its nuclear export signal (NES)^18,19^ (Fig. 1a,b). The snRNA export complex then translocates to the cytoplasm where it disassembles upon GTP hydrolysis triggered by Ran activating factors^18,19^ (Fig. 1b). Additional control of this process involves the nuclear phosphorylation by casein kinase 2 (CK2) of multiple serines in the phosphorylation site cluster 2 (ST2) of PHAX (Fig. 1a), which is essential for stable complex formation and export^18,20^. In the cytoplasm, snRNA export complex disassembly is further promoted by PHAX dephosphorylation by protein phosphatase 2 (refs. ^18,20^). The PHAX–CRM1 pathway has also been implicated in the nuclear export of a small class of m^7^G-capped pre-microRNAs^21^ as well as histone H2AX mRNA^22^.

Human PHAX consists of 394 amino acid residues and is predicted to be mostly intrinsically disordered except for its RNA binding domain (RBD)^23,24^ (Fig. 1a). PHAX can form a CBCAP complex with CBCA^25^, and in vitro, the N-terminally located ARM motif of PHAX binds to the effector domain of ARS2 (refs. ^7,26^). However, in the presence of ARS2, PHAX is unable to directly bind to the CBC owing to steric competition by ARS2 (ref. ^7^). This suggests that ARS2 functions as a gatekeeper, preventing RNA-bound CBC to commit to RNA export until appropriate conditions are met. The cryo-electron microscopy (cryo-EM) structure of the CBC–PHAX (CBCP) complex revealed that the W118-containing helix of PHAX, as well as its C-terminus, directly bind to the CBC^7^. Indeed, the PHAX C-terminus binds in the same groove between CBP20 and CBP80 that also accommodates the ARS2 C-terminus^7^, consistent with a possible competition between ARS2 and PHAX for CBC binding.

Here, we reconstitute in vitro the core snRNA export complex, comprising capped RNA, CBC, phosphorylated PHAX and CRM1–RanGTP, and we confirm that PHAX phosphorylation stabilizes the complex. The cryo-EM structure at 2.45 Å resolution shows how synergistic interactions allow PHAX to simultaneously bridge the CBC and CRM1 while reinforcing dinucleotide cap recognition and CRM1 binding. Unexpectedly, we also find that a conserved two-residue motif of PHAX binds to a distant region of CRM1, suggesting that the adjacent phosphorylated ST2 cluster of PHAX could interact with a prominent basic patch of RanGTP. The Ran basic patch also binds the RNA component of export complexes for transfer RNA (tRNA), pre-microRNA (miRNA) and human immunodeficiency virus (HIV) Rev response element (RRE) with their respective export adaptors EXPO-T, EXPO-5 and CRM1 (refs. ^27–29^). Thus, PHAX phosphorylation may not only increase export complex stability but also enable favorable competition with other CRM1 RNA cargoes, in a potentially regulatable fashion^18^.

## Results

### Cryo-EM structure of the snRNA export complex

**Extended Data Fig. 1:**
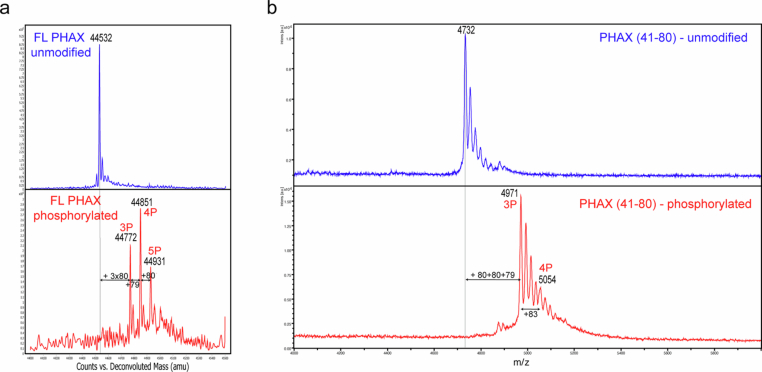
PHAX phosphorylation analysis. Masses of CK2-treated FL PHAX and PHAX^41–80^ were assessed using mass spectrometry (MS). **a**. FL PHAX was analyzed by LC-ESI-MS (liquid chromatography-electrospray). Data are presented after deconvolution of *m/z* (mass-to-charge ratio) to masses (expressed in Da). Spectra of FL PHAX before and after phosphorylation were compared. The full-length protein has between 3 and 5 phosphorylated sites (3 P, 4 P and 5 P). LC-ESI-MS data were collected using Mass hunter (v. 11.0, Agilent Technologies) and processed using Bioconfirm (v. 12.01, Agilent Technologies) and GPMAW (v. 7.00b2, Lighthouse Data, Denmark) software. **b**. PHAX (41–80) was analyzed by MALDI-MS (Matrix Assisted Laser Desorption Ionisation-mass spectrometry). MALDI-MS determines *m/z* values of singly charged PHAX^41–80^ ions. The *m/z* difference between the untreated PHAX (41–80) and the phosphorylated one indicates that PHAX^41–80^ carries 3 phosphorylated sites (3 P). An additional site (4 P) may be present. Peaks that are not labeled are due to adducts with non-volatile molecules (such as Na). MALDI-MS data were collected using flexControl™ (v.3.4, Bruker Daltonik) and processed using flexAnalysis™ (v. 3.4, Bruker Daltonik).

**Extended Data Fig. 2:**
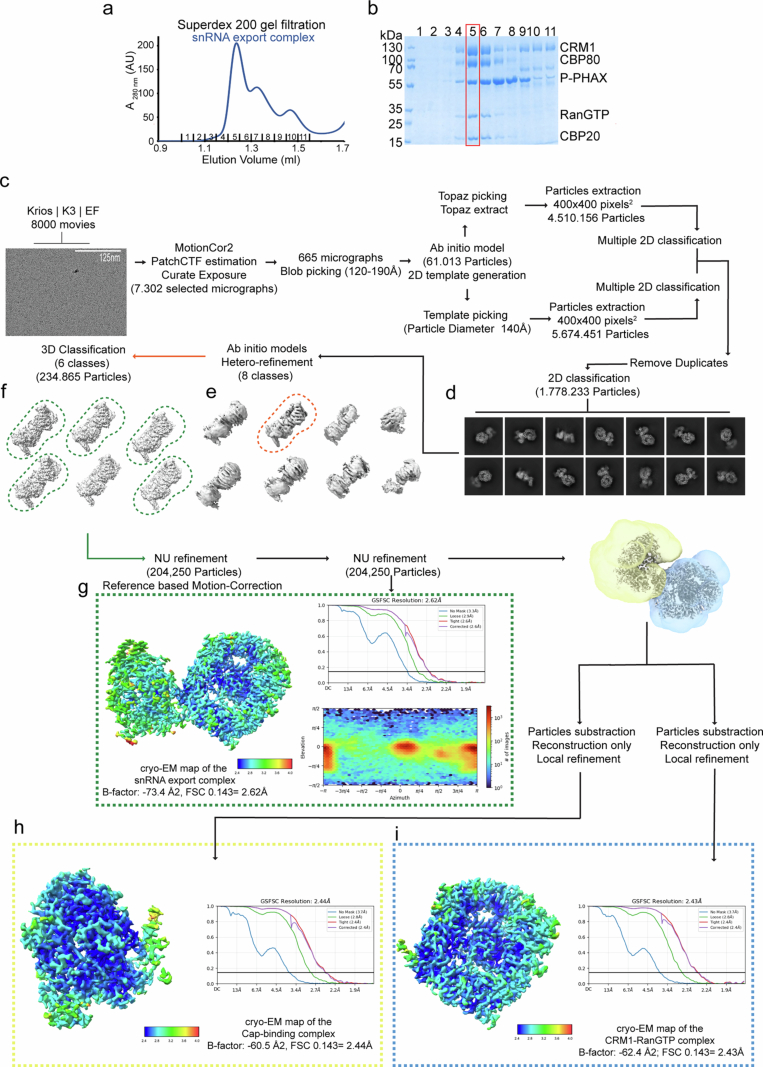
Cryo-EM image processing strategy used to obtain the structure of the snRNA export complex. **a,b**. Superdex 200 gel filtration elution profile and SDS-PAGE analysis of fraction 1–12 of the snRNA complex reconstituted with capped 14nt RNA. The complex eluted in fraction 5 used for grid preparation is highlighter with a red rectangle in **b**. **c**. Representative cropped micrograph (Scale bar = 150 nm). **d**. 2D class averages. **e,f**. 3D class averages. **g-i**. Views of each local resolution sharpened EM map are shown. Fourier shell correlation curves (FSC) are displayed. Source data

To understand the molecular details underlying the role of PHAX within the snRNA export complex, we determined its structure. The export complex was reconstituted with purified CBC, in vitro CK2-phosphorylated PHAX, exportin CRM1, the catalytic mutant of Ran (Q69L) bound to GTP and an in vitro transcribed and capped U1 snRNA (164 nt). Phosphorylation of PHAX was verified by gel shift assays and mass-spectrometry revealing up to five phosphorylation sites (four within ST2) (Extended Data Fig. 1a,b). The complex was purified using size exclusion chromatography where all the components co-eluted in a peak with the expected apparent stoichiometry (Fig. 1c,d). An equivalent complex was also reconstituted with a short capped RNA containing 14 nucleotides (m^7^GpppAAUCUAUAAUAGCA), suggesting that specific snRNA features might not be required for complex formation in vitro (Fig. 1e,f and Extended Data Fig. 2a,b). As expected, when PHAX is not phosphorylated, the export complex formed with markedly lower efficiency (Fig. 1e–g). A preliminary cryo-EM characterization of the two complexes, containing either the U1 snRNA or the short RNA, resulted in essentially identical reconstructions, where the bulk of the RNA was not visible. Here, we describe the structure containing the short capped RNA, which was obtained at an overall resolution of 2.8 Å, limited by slight flexibility between the CRM1–RanGTP and CBC–PHAX moieties. Focused refinement on each moiety separately improved the resolution to around 2.45 Å (Fig. 1h,i, Table 1, Extended Data Fig. 2 and Supplementary Fig. 1).

**Table 1:** Cryo-EM data collection, refinement and validation statistics

	snRNA export complex (EMD-52115), (PDB 9HFL)		
**Data collection and processing**			
Magnification	105,000×		
Voltage (kV)	300		
Electron exposure (e^–^ Å^−^^2^)	40.5		
Defocus range (μm)	−1.0 to −2.2		
Pixel size (Å)	0.84		
Symmetry imposed	–		
Initial particle images (no.)	1,778,233		
Final particle images (no.)	204,250		
	**Complete, map**	**Focused map, CBC**	**Focused map, CRM1**
Map resolution (Å)	2.62	2.44	2.43
FSC threshold	0.143	0.143	0.143
Map resolution range (Å)	2.25–3.85	2.05–3.50	2.10–4.01
**Refinement**	**Composite focused maps**		
Initial model used (PDB code)	8PNT, 3NC1		
Model resolution (Å)	2.41		
FSC threshold	0.143		
CC (mask)	0.8246		
Map sharpening *B* factor (Å^2^)	−73.4	−60.5	−62.4
Model composition			
Nonhydrogen atoms	17,802		
Protein residues	2159		
Ligands	3 (m^7^GpppA, GTP, Mg)		
Waters	32		
*B* factors (Å^2^)			
Protein	54.05		
Ligand	39.30		
Waters	39.70		
R.m.s. deviations			
Bond lengths (Å)	0.002		
Bond angles (°)	0.428		
**Validation**			
MolProbity score	1.38		
Clashscore	3.30		
Poor rotamers (%)	2.19		
Ramachandran plot			
Favored (%)	98.09		
Allowed (%)	1.91		
Disallowed (%)	0.00		

The cryo-EM map covers all the complex components (Fig. 1h,i) with CBC and the CRM1–RanGTP forming two lobes, each adopting essentially the same structures as reported previously^8,30^. The two lobes are connected by PHAX. In our previous structure of CBC–PHAX bound to a 12-nt capped RNA, only residues 114–132 of PHAX were visible, forming a short tryptophan-containing helix interacting with CBP80 (ref. ^7^) (Fig. 1a). In the presence of CRM1–RanGTP, a larger region of PHAX is folded, encompassing residues 112–162. The W118-containing PHAX helix is extended to include the PHAX NES, which binds to CRM1, thus rigidly bridging the RNA-binding CBC to the export factor. The downstream segment of PHAX (residues 140–162) is also structured, forming contacts with both CBP20 and the RNA cap structure. Finally, a second region of PHAX, spanning N-terminal residues 55–60, binds between HEAT repeats 5 and 6 of CRM1. Only the cap structure of the RNA is well-defined, indicating flexibility of the rest of the RNA molecule. Consistent with this, the RBD domain of PHAX is also not visible. The structure thus reveals the key role of PHAX^112–162^ in recruiting CRM1–RanGTP to the CBC-bound capped RNA.

### PHAX mediates the interaction between the CBC and CRM1

**Fig. 2:**
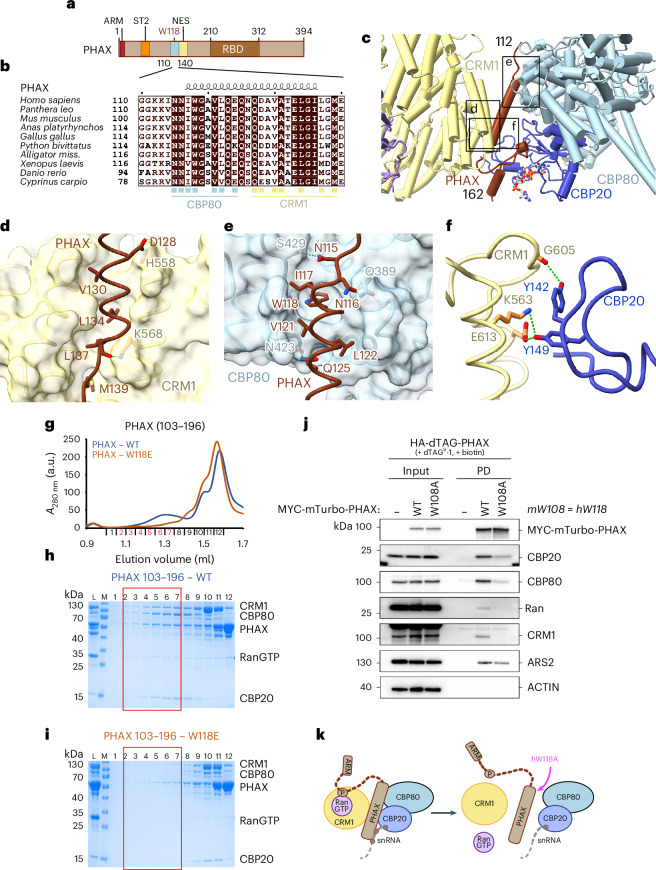
PHAX-mediated contacts between CBC and CRM1. **a**, Schematic representation of the PHAX domain structure. ARM, ARS2 recognition motif; ST2, phosphorylation site cluster 2; NES, nuclear export signal; RBD, RNA binding domain. **b**, Sequence alignment of PHAX proteins covering the CBC-binding and NES regions. Identical residues are in brown boxes. Blue squares indicate residues involved in the interaction with CBC, and yellow squares show residues interacting with CRM1. **c**, Ribbon representation of the CBC–CRM1 interactions mediated by PHAX and CBP20. The three black rectangles position the close-up views shown in **d**–**f**. **d**, Details of the interactions between the PHAX helix (residues 117–136, dark brick) and CRM1 shown as surface (yellow). The NES residues of PHAX insert into hydrophobic pockets on CRM1. Several charged contacts with CRM1 are shown. **e**, Details of the hydrophobic and charged interactions between the PHAX helix (residues 117–136, dark brick) and CBP80 shown as surface (light blue). **f**, Molecular details of the interactions between the CRM1 (yellow) and CBP20 (dark blue). **g**, Overlay of Superdex 200 gel filtration elution profiles of the snRNA complex reconstitutions using either WT or W118E mutant of MBP-PHAX^103–196^ and a short capped 14-nt RNA. The WT complex elutes in fractions highlighted in red. **h**,**i**, SDS-PAGE analysis of fractions 1–12 of the gel filtration elution profiles of WT (**h**) and W118E mutant (**i**) of MBP-PHAX^103–196^ shown in **g**. The red rectangle shows the fractions containing the snRNA export complex. In **i**, MBP-PHAX^103–196^ W118E is unable to form the complex with CBC and CRM1–RanGTP. L, input sample loaded onto the column; M, Mw marker. **j**, Western blotting analysis of streptavidin pulldowns from lysates of HA-dTAG-PHAX mES cells stably expressing MYC-mTurbo tagged PHAX^WT^ or PHAX^W108A^ with the parental cell line (−) serving as a negative control. Cells were treated with dTAG^V^-1 for 4 h to deplete endogenous HA-dTAG-PHAX protein and treated with biotin for a further 4 h to induce PL. Input (left) and streptavidin-enriched pulldown (PD; right) samples were probed with antibodies against MYC, CBP20, CBP80, Ran, CRM1 and ARS2 with ACTIN serving as an input loading control. **k**, A schematic model of the impact of hW118A mutation on the snRNA export complex assembly. Source data

PHAX residues 117–136 form a long helix that interacts with both the CBC and CRM1 (Fig. 2a–c). Its C-terminal half includes the NES whose residues dock into corresponding hydrophobic pockets of the NES-binding cleft of CRM1 (Fig. 2c,d and Supplementary Fig. 1a). While V130, L134, L137 and M139 bind CRM1 in a canonical way, the first residue of the PHAX NES sequence (Φ0) is a conserved glutamine (Q127) rather than a hydrophobic residue. This may weaken the PHAX NES-CRM1 interaction compared with a canonical NES^31^ (Fig. 2b). In addition, PHAX residues D128 and L137 form hydrogen bonds with CRM1 H558 and K568 (Fig. 2d).

The N-terminal half of the same PHAX helix binds to CBC as reported in our previous CBC–PHAX–RNA structure^7^, with the W118 residue inserting into the conserved hydrophobic pocket on CBP80 (Fig. 2c,e and Supplementary Fig. 1b). However, within the export complex, several extra contacts occur, including hydrogen bonds formed between N115, N116 and Q125 of PHAX and S429, Q389 and N423 of CBP80 (Fig. 2e). Whereas in the binary CBC–PHAX complex the extreme C-terminus of PHAX (residues 390–394) bound in the groove between CBP80–CBP20 (ref. ^7^), in the export complex structure the groove is empty. CBC and CRM1 also directly interact via several hydrogen bonds between Y142 and Y149 of CBP20 and K563, G605 and E613 of HEAT repeats 12 and 13 of CRM1 (Fig. 2f).

**Extended Data Fig. 3:**
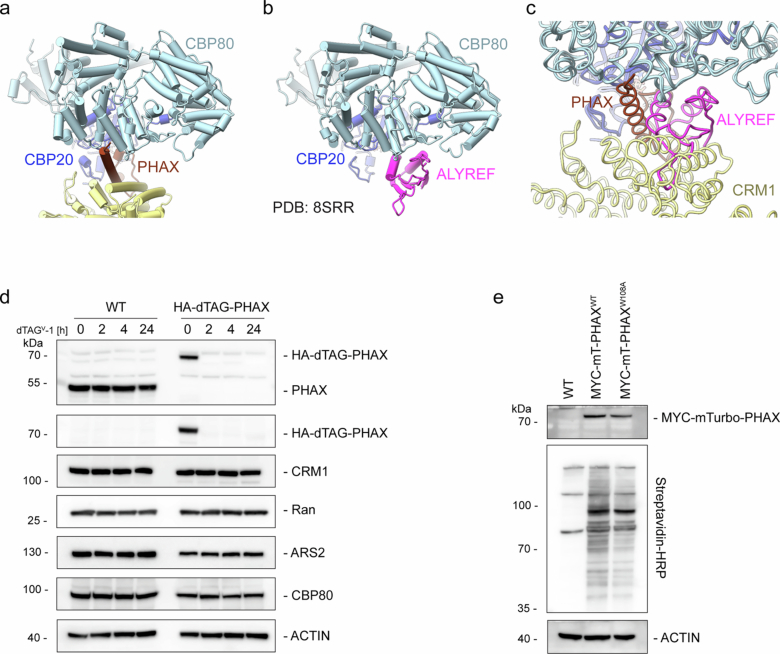
CBC Binding of ALYREF is incompatible with binding of PHAX and CRM1. **a-c**. Comparison of CBC interactions with PHAX and CRM1 (**a**) with the CBC contacts with ALYREF (**b**) (PDB:8SRR, shown in magenta). The two structures were superimposed using CBC (**c**). The binding of ALYREF to the CBC is incompatible with that of PHAX and CRM1. **(d)** Western blot analysis of lysates from WT or HA-dTAG-PHAX mES cells harvested at indicated time points (hours) following dTAG^V^-1 treatment. Membranes were probed with antibodies against PHAX, HA, CRM1, Ran, ARS2 and CBP80. ACTIN was used as a loading control. **(e)** Western blot analysis of lysates from WT control or HA-dTAG-PHAX mES cells expressing MYC-miniTurbo(mT)-PHAX proteins. Cells were treated with dTAG^V^-1 to deplete endogenous dTAG-PHAX and biotin was added for 6 h to induce proximity labeling. Membranes were probed with MYC and ACTIN to assess protein expression and Streptavidin-HRP to assess biotinylation efficiency of mTurbo-tagged proteins. Source data

PHAX, NELF-E, NCBP3 and ZC3H18 all recognize the same surface on the CBC via their conserved tryptophan-containing helices^7^. Furthermore, the mRNA export factor ALYREF interacts with a neighboring CBC surface^32^. The comparison of our snRNA export complex structure with that of CBC bound to the RRM domain of ALYREF revealed that ALYREF binding to CBC is incompatible with the CBC binding to PHAX and CRM1 (Extended Data Fig. 3a–c). This mutual exclusivity may thus contribute to directing snRNAs and mRNAs into distinct pathways for nuclear export.

### CBC–PHAX interaction is critical for snRNA complex assembly

Since the PHAX core region observed in the cryo-EM structure includes residues 112–162, we tested whether a corresponding PHAX fragment is sufficient to form the export complex. Indeed, PHAX^103–196^ lacking the upstream CRM1-binding sequence (residues 55–60, see below), the ST2 phosphorylated region and the downstream RBD still formed the complex, albeit with lower CRM1 stoichiometry than full-length phosphorylated PHAX (Fig. 2g,h). PHAX W118E mutation is sufficient to disrupt PHAX^103–196^ binding to the CBC^7^. Importantly, this mutation also impeded snRNA export complex formation (Fig. 2g,i), showing that the interaction between the W118-containing helix and the CBC remains critical within the complete export complex.

To test the importance of the PHAX W118-containing helix in the export complex formation in vivo, we utilized a proximity labeling (PL) pulldown approach to explore the recruitment of proteins to wildtype (WT) or mutated PHAX. We first tagged endogenous PHAX alleles in mouse embryonic stem (mES) cells with a 2xHA-FKBP-V (HA-dTAG) degron tag to allow for the rapid depletion of HA-dTAG-PHAX proteins upon the addition of the dTAG^V^-1 ligand. This was efficiently achieved following 2 h of dTAG^V^-1 treatment, with no consequential effects on steady state levels of CBCA or CRM1–Ran proteins (Extended Data Fig. 3d). We then utilized the PiggyBac transposase system^33^ to stably introduce MYC-mini-Turbo (mTurbo) tagged PHAX^WT^ or PHAX^W108A^ (mouse equivalent of W118) into the HA-dTAG-PHAX cells. The mTurbo PL system allows for in vivo biotinylation of proteins in the vicinity (1–10 nm) of the fusion protein upon the addition of biotin to media. Following depletion of the endogenous HA-dTAG-PHAX protein and subsequent addition of biotin, MYC-mTurbo-PHAX^WT^ and PHAX^W108A^ efficiently biotinylated proteins when compared with cells lacking mTurbo (Extended Data Fig. 3e). Having validated the system, we proceeded to purify in vivo biotinylated proteins from cells expressing MYC-mTurbo-PHAX^WT^ or PHAX^W108A^ by streptavidin-enrichment.

Western blotting analysis revealed that PHAX^WT^ pulldowns were enriched for CBC, ARS2, CRM1 and Ran proteins compared with an untagged control (Fig. 2j). However, the PHAX^W108A^ mutant showed a marked decrease in CBC proteins and a complete loss of CRM1 and Ran (Fig. 2j,k). These results demonstrate that the W118-mediated interaction between PHAX and CBC is crucial for efficient formation of the snRNA export complex (Fig. 2k).

### PHAX is intimately involved in RNA cap recognition

**Fig. 3:**
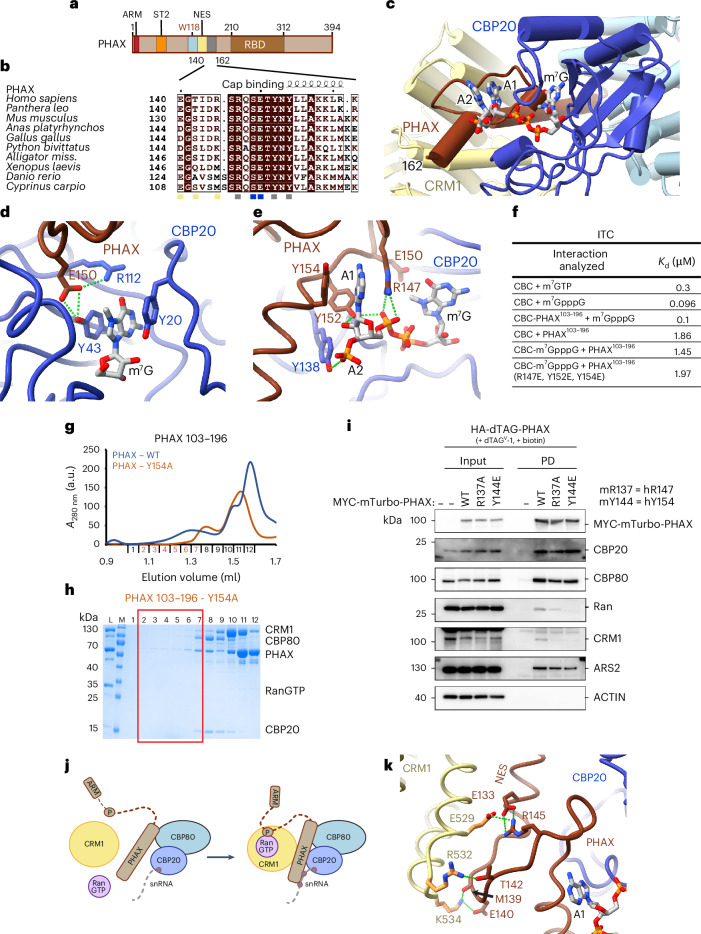
RNA recognition by PHAX. **a**, Schematic representation of the PHAX domain structure. ARM, ARS2 recognition motif; ST2, phosphorylation site cluster 2; NES, nuclear export signal; RBD, RNA binding domain. **b**, The sequence alignment of PHAX proteins covering the cap- and CRM1-binding regions. Identical residues are in brown boxes. Blue squares indicate residues involved in the interaction with cap, yellow squares show residues interacting with CRM1 and gray squares show residues binding RNA. **c**, Ribbon representation of the RNA recognition by the snRNA export complex. **d**, Details of the interactions of PHAX E150 with key CBP20 cap-binding residues. **e**, Details of the specific recognition of the first-transcribed residue by PHAX. **f**, Summary of the ITC measurements of binding affinities (expressed as the dissociation constant, *K*_d_) between CBC, PHAX^103–196^ and the cap structure. **g**, Overlay of Superdex 200 gel filtration elution profiles of the snRNA complex reconstitutions using either WT or Y154A mutant of MBP-PHAX^103–196^ and a short capped 14-nt RNA. The WT complex elutes in fractions highlighted in red. **h**, SDS-PAGE analysis of fractions 1–12 of the gel filtration elution profiles shown in **g**. MBP-PHAX^103–196^ Y154A is unable to form the complex with CBC and CRM1–RanGTP. The fractions corresponding to the WT complex elution peak are highlighted with the red rectangle. L, input sample loaded onto the column; M, Mw marker. **i**, Western blotting analysis of streptavidin pulldowns from lysates of HA-dTAG-PHAX mES cells stably expressing MYC-mTurbo tagged PHAX^WT^, PHAX^R137A^ or PHAX^Y144E^ as described in Fig. 2j. **j**, A schematic model of snRNA export formation based on RNA- and CRM1–RanGTP-triggered folding of PHAX. **k**, Details of direct interactions between CRM1 and the PHAX region downstream NES. Source data

**Extended Data Fig. 4:**
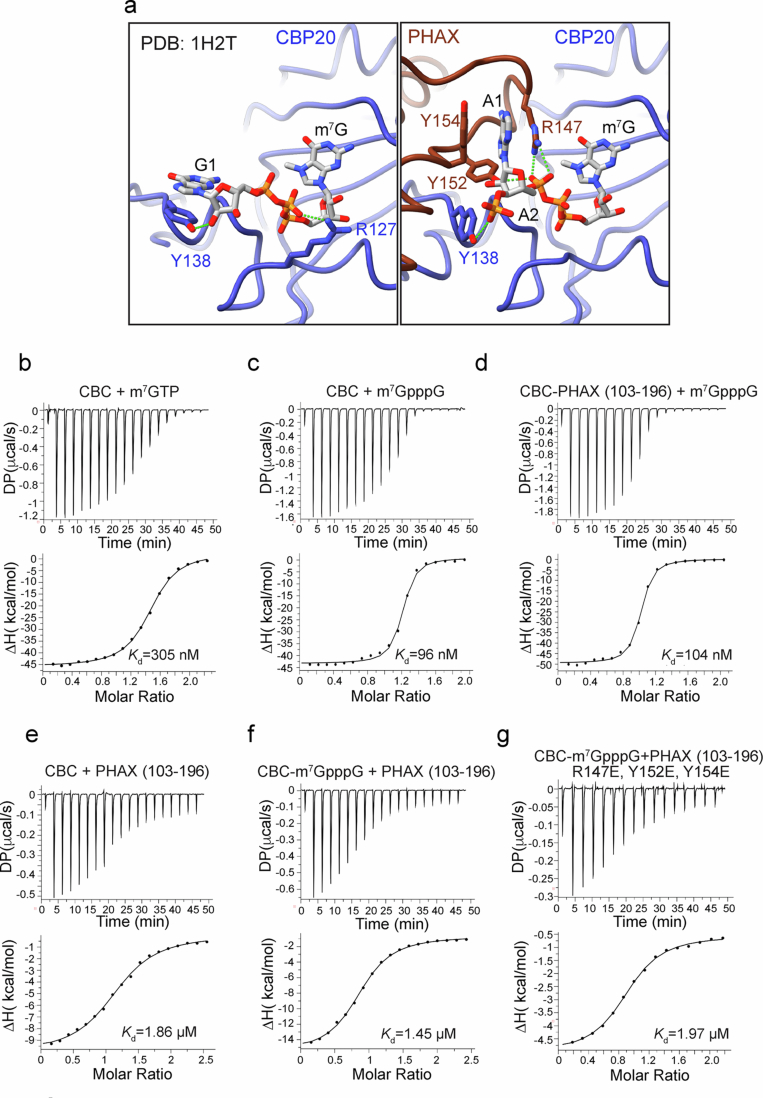
Characterization of RNA recognition by PHAX. **a**. Comparison of the molecular details of RNA cap binding by CBC and CBC-PHAX. **b**. ITC measurement of the interaction affinity between CBC and of m^7^GTP. **c,d**. ITC measurements of the interaction affinity between CBC and cap analog (m^7^GpppG) in the absence or presence of PHAX^103–196^. **e,f**. ITC measurements of the interaction affinity between CBC and PHAX^103–196^ in the absence or presence of m^7^GpppG. **g**. ITC measurement of the interaction affinity between CBC-m^7^GpppG and the R147E, Y152E, Y154E mutant of PHAX^103–196^.

The snRNA export complex structure reveals that conserved PHAX residues 140–162, downstream of the PHAX^116–136^ bridging helix, engage in specific contacts with the RNA cap structure, CBP20 and CRM1 (Fig. 3a–c and Supplementary Fig. 1c). Details of m^7^GTP cap-moiety recognition by CBC alone are well established^8,10^ (Extended Data Fig. 4a) and in the presence of PHAX, the methylated guanosine and the α-phosphate of the cap are bound by CBP20 as previously reported. However, we found that conserved E150 of PHAX inserts into the cap-binding site, forming hydrogen bonds with R112 and Y43 of CBP20. Since these two residues are key for the specific interaction with m^7^G, PHAX probably strengthens cap recognition by CBP20 (Fig. 3d).

A crucial difference from cap binding by CBC alone is the strong additional binding of PHAX to the first-transcribed RNA nucleotide. In the CBC-cap analog (m^7^GpppG) complex structure^8^, the first-transcribed nucleotide (here G1) stacks with the Y138 residue of CBP20 (Extended Data Fig. 4a). Moreover, studies employing the Y138A mutant showed that this residue confers additional binding affinity for dinucleotide cap analogs^34^. However, in the presence of PHAX, the first-transcribed nucleotide (here A1) is shifted away from CBP20 and forms multiple interactions with PHAX (Fig. 3e and Extended Data Fig. 4a). Importantly, A1 is sandwiched between PHAX R147 and Y154. In addition, R147 and Y152 interact with the phosphate Pγ and the ribose moiety of A1 (Fig. 3e). This results in a slight movement of Pβ and Pγ, and loss of a hydrogen bond between Pβ and CBP20 R127 (Extended Data Fig. 4a). Y138 of CBP20 now binds the phosphate group of the following nucleotide (Fig. 3e and Extended Data Fig. 4a).

### Cap recognition by PHAX requires the presence of CRM1–RanGTP

The snRNA export complex structure reveals that, in the presence of CRM1–RanGTP, PHAX contributes strongly to RNA cap binding (Fig. 3c–e). However, in a cryo-EM structure of full-length PHAX bound to the CBC and a 12-nt capped RNA, no contacts of PHAX with the cap were visible^7^. Thus, PHAX might recognize RNA only when residues 140–162 are stabilized by the presence of CRM1–RanGTP. To address this issue, we used isothermal calorimetry (ITC) to measure the binding affinity (expressed as the dissociation constant, *K*_d_) between CBC, PHAX^103–196^ and the cap structure. We found that CBC binds m^7^GTP with a *K*_d_ of 300 nM (Fig. 3f and Extended Data Fig. 4b). For the cap analog m^7^GpppG, the measured *K*_d_ was 96 nM (Fig. 3f and Extended Data Fig. 4c). This higher affinity for the cap analog is consistent with previous observations that the first-transcribed G stacks with Y138 of CBP20 (refs. ^8,10^). Importantly, when CBC was in complex with PHAX^103–196^—which bears residues sufficient for CBC and cap binding—the affinity for the cap analog remained unchanged. This suggests that PHAX does not contribute to RNA binding in this context, as observed structurally (Fig. 3f and Extended Data Fig. 4d). Similarly, the presence of the second nucleotide of the cap did not notably increase the affinity of CBC to PHAX^103–196^, and the triple mutation of the three key A1-binding residues of PHAX (R147E, Y152E and Y154E) had essentially no impact on PHAX binding to the CBC in the presence of the cap analog (Fig. 3f and Extended Data Fig. 4e–g). Thus, in the absence of CRM1–RanGTP, the addition of PHAX to the CBC does not result in a measurable binding affinity change to the cap structure, suggesting that the corresponding regions of PHAX are disengaged.

### Cap binding by PHAX is required for export complex formation

Next, we investigated the role of the capped RNA in snRNA export complex formation. While the structure suggests that CRM1–RanGTP-triggered folding of PHAX^140–162^ would enhance the recognition of capped RNA by PHAX (although we have not shown this directly by binding studies), reciprocally, it also indicates that cap-dependent folding of PHAX^140–162^ is probably required for efficient interaction between CBC–PHAX and CRM1–RanGTP.

**Extended Data Fig. 5:**
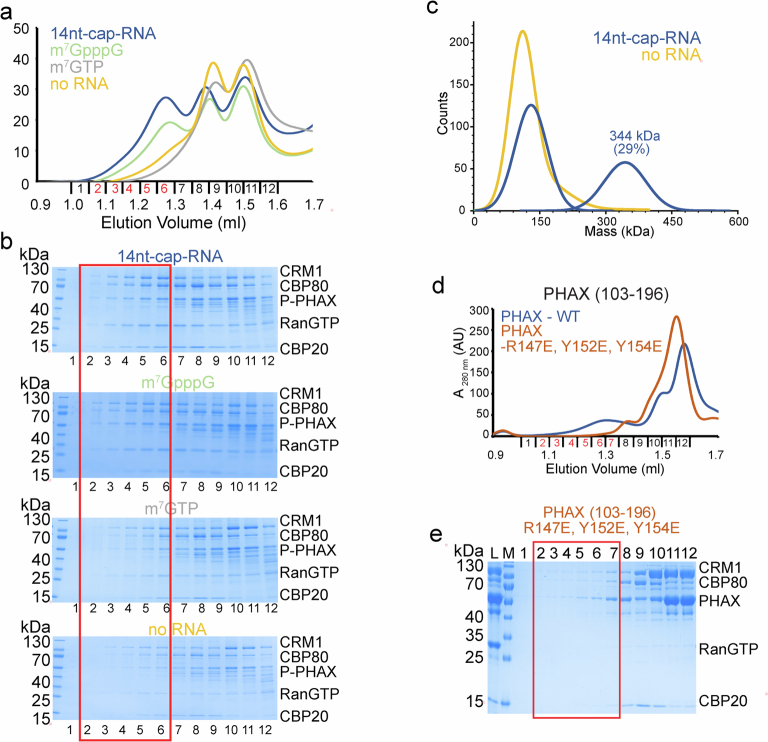
RNA is required for snRNA complex formation. **a**. Overlay of Superdex 200 gel filtration elution profiles of the snRNA complex reconstitutions with different lengths of RNA. **b**. SDS-PAGE analysis of fractions 1–12 of the Superdex 200 gel filtration elution profiles shown in **a**. **c**. Mass photometry analysis of the snRNA complex formed with phosphorylated FL PHAX in the presence or absence of 14nt capped RNA. The measured molecular mass the full complex and the percentage of corresponding observations is indicated. **d**. Overlay of Superdex 200 gel filtration elution profiles of the snRNA complex reconstitutions with using either WT or the R147E, Y152E, Y154E mutant of MBP-PHAX^103–196^ and a short capped 14nt RNA. The WT complex elutes in fractions highlighted in red. **e**. SDS-PAGE analysis of fractions 1–12 of the gel filtration elution profile shown in **c**. MBP-PHAX^103–196^ R147E, Y152E, Y154E is unable to form the complex with CBC and CRM1-RanGTP. The fractions corresponding to the WT complex elution peak are highlighted with red rectangle. Source data

Indeed, using size exclusion chromatography and mass photometry, we find that snRNA export complex formation depends on the presence of capped RNA, with at least a dinucleotide cap being required (Extended Data Fig. 5a–c). Furthermore, the triple mutation, R147E, Y152E and Y154E, within the cap-binding region of PHAX^103–196^ disrupted the complex (Extended Data Fig. 5d,e). The same negative impact was observed even for the single Y154A mutation (Fig. 3g,h). Our parallel in vivo analysis, employing PHAX R137A and Y144E mutations (mouse equivalents of R147A or Y154E), showed markedly reduced PL of PHAX with CRM1 and Ran, while CBC and ARS2 labeling remained unchanged (Fig. 3i). We therefore conclude that the strong interaction of the first transcribed nucleotide with PHAX is required for efficient recruitment of CRM1 and RanGTP to the complex.

SnRNA export complex formation thus requires a synergistic, mutually dependent assembly of all its components. It has been established that efficient NES recognition by CRM1 requires the presence of Ran-GTP (ref. ^35^) and that efficient cap binding by CBP20 depends on CBP80 (ref. ^8^). PHAX is key for bridging these two modules and its proper conformation depends on the presence of CRM1–RanGTP, CBC and capped RNA (Fig. 3j). An important role in the structuring of PHAX is probably also played by residue R145, which connects the PHAX region bound to RNA and CBP20 to the PHAX helix and CRM1 through charged interactions with PHAX E133 and CRM1 E529 (Fig. 3k). PHAX residues M139, E140 and T142 also form additional contacts with CRM1 residues R532 and K534, strengthening the NES–CRM1 interface.

### The role of PHAX phosphorylation

**Fig. 4:**
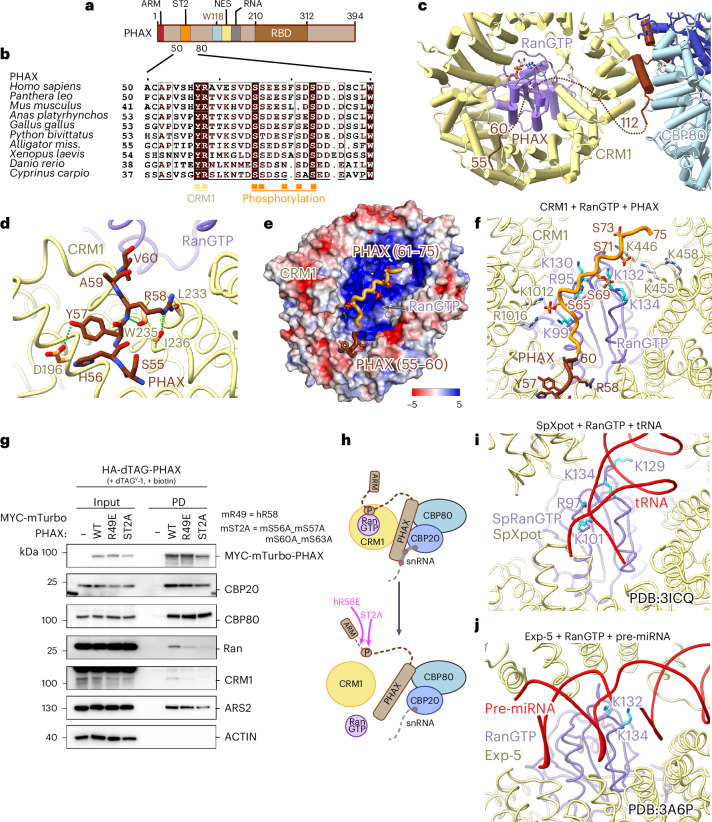
PHAX N-terminus connects CRM1–RanGTP. **a**, A schematic representation of the PHAX domain structure. ARM, ARS2 recognition motif; ST2, phosphorylation site cluster 2; NES, nuclear export signal; RBD, RNA binding domain. **b**, Sequence alignment of PHAX proteins covering the CRM1-binding and phosphorylation regions. Identical residues are shown in brown boxes. Orange squares indicate potential phosphorylated residues and yellow squares show residues interacting with CRM1. **c**, A ribbon representation of the interaction between CRM1 and the N-terminus of PHAX. **d**, Details of the interactions between PHAX^56–60^ and CRM1 showing charged contacts between the two proteins. **e**, Electrostatic surface potential representation of the CRM1–RanGTP complex calculated using APBS^43^ and visualized in PyMOL^44^. The color gradient range is from red (−5 *k*^B^*T* per elementary charge) to blue (+5 *k*^B^*T* per elementary charge). PHAX^55–60^ is shown in brown as sticks and the AlphaFold3 model of following the ST2 region is in orange with phosphorylated serines as sticks binding over the basic surface of Ran. **f**, Illustration of the CRM1–RanGTP–PHAX complex. PHAX^55–60^ is shown in brown as sticks. The AlphaFold3 model of following the ST2 region is shown in orange with phosphorylated serines as sticks positioned in proximity of positively charged residues of the basic surface of Ran. **g**, Western blotting analysis of streptavidin pulldowns from lysates of HA-dTAG-PHAX mES cells stably expressing MYC-mTurbo tagged PHAX^WT^, PHAX^R49E^ or PHAX^ST2A^ as described in Fig. 2j. **h**, A schematic model of the impact of hR58E and ST2A mutation on the snRNA export complex assembly. **i**, Contacts between the basic surface of *S. pombe* RanGTP and tRNA within the tRNA nuclear export complex^27^. **j**, Contacts between the basic surface of RanGTP and pre-miRNA within the pre-miRNA nuclear export complex^28^. Source data

**Extended Data Fig. 6:**
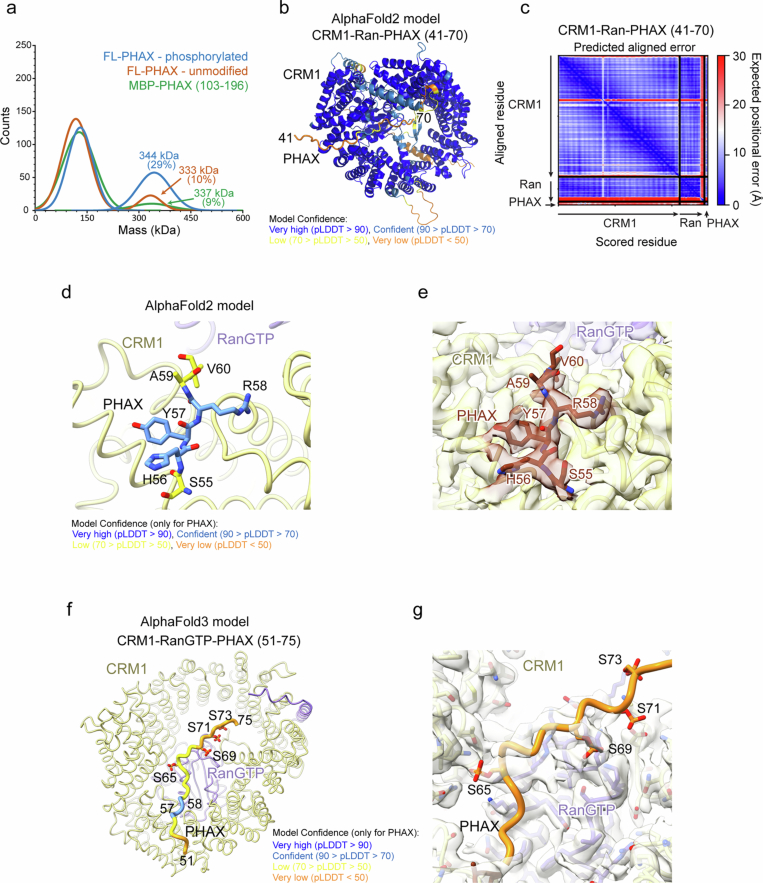
CRM1-Ran-PHAX structure prediction. **a**. Mass photometry analysis of the snRNA complex formed with phosphorylated FL PHAX, unmodified FL PHAX and MBP-PHAX^103–196^ in the presence of 14nt capped RNA. The measured molecular mass of the full complex and the percentage of corresponding observations is indicated. **b**. AlphaFold2 predicted structure of CRM1-Ran-PHAX^41–70^ colored according to pLDDT. **c**. Predicted aligned error (PAE) plot for the CRM1-Ran-PHAX^41–70^ model. **d**. AlphaFold2 model of CRM1-Ran-PHAX^41–70^. Only residues 55–60 of PHAX are shown as sticks. Only PHAX is colored according to pLDDT. CRM1 is shown in yellow. **e**. Cryo-EM map and details of the interactions between CRM1 and the PHAX^56–60^. **f**. AlphaFold3 model of CRM1-Ran-PHAX^1–103^ with phosphorylation on serines 65,69,71 and 73. Only residues 55–75 of PHAX are shown. Only PHAX is colored according to pLDDT. CRM1 is shown in yellow and Ran GTP in violet. **g**. Extra electron density, appearing at low contour level, that partially overlaps with positions of phosphorylated serines predicted by AlphaFold3.

PHAX contains eight putative phosphorylation clusters, but only the second cluster (ST2, residues 64–76) (Fig. 4a,b) is essential for snRNA export complex assembly and export^20^. Gel filtration experiments with unphosphorylated PHAX confirmed that the snRNA export complex forms with markedly lower efficiency (Fig. 1e–g). Similarly, PHAX^103–196^, lacking ST2 but encompassing the region at the CBC–CRM1 interface, can still form the snRNA export complex, albeit less efficiently (Fig. 2g–i). Equivalent results were obtained using mass photometry (Extended Data Fig. 6a). How the phosphorylation of ST2 enhances complex assembly and promotes export is therefore an important outstanding question.

Although PHAX^112–162^ is the key region bridging CBC–RNA to CRM1–RanGTP, the cryo-EM map also exhibited extra density between CRM1 HEAT repeats 5 and 6. Interestingly, AlphaFold2^36^ predictions of the CRM1–RanGTP–PHAX complex show, with high confidence, binding of PHAX^56–60^ to exactly this position (Extended Data Fig. 6b–d). Two confidently predicted and conserved PHAX residues, Y57 and R58 (Fig. 4b), engage in several charged contacts with CRM1 (Fig. 4c,d), fitting very well into the extra cryo-EM density (Extended Data Fig. 6e). Intriguingly, PHAX^56–60^ is immediately upstream of the ST2 phosphorylation cluster, which also includes a number of acidic residues (64-DSSEESFSDSDDD) (Fig. 4a,b), typical of a multiple CK2 phosphorylation site^37,38^. While AlphaFold3^39^ does not make a unique, confident prediction of the position of the phosphorylated ST2 Ser residues, all models displayed the ST2 peptide (which is pinned on one side by the Y57-R58 binding to CRM1) lying on a strongly basic surface of CRM1–RanGTP (Fig. 4e,f and Extended Data Fig. 6f). Bearing in mind that ST2 phosphorylation may be heterogeneous, this would be consistent with a possibly ‘fuzzy’, electrostatically driven interaction between ST2 and this region of CRM1–RanGTP. As an indication of how the pSer (and other acidic residues in ST2 of PHAX) could interact with the basic surface formed by both CRM1 and Ran, pSer65 is spatially close to CRM1 residues K1012, R1016 and Ran residue K99. Moreover, pSer69 and 71 are close to Ran K132, K134. Additionally, Ran R95, K130 and CRM1 K446, K455 and R458 would be in the vicinity of the ST2 peptide (Fig. 4f). Indeed, further examination of the cryo-EM map showed weak extra density to support this suggestion (Extended Data Fig. 6g).

The importance of both PHAX^55–60^ binding to CRM1 and ST2 phosphorylation for snRNA export complex formation was tested in vivo, employing mutations of equivalent residues in the mouse PHAX protein. These mutants (mR49E and mS56A-S57A-S60A-S63A) markedly reduced recruitment of CRM1 and RanGTP to PHAX, while having little impact on CBCA association (Fig. 4g,h). We therefore conclude, that the interaction between PHAX^55–60^ and CRM1, which probably enables proper positioning of the phosphorylated ST2 region on the basic surface on RanGTP, is required for efficient CRM1–RanGTP recruitment to the complex. Within tRNA, pre-miRNA and HIV RRE export complexes, the basic surface of Ran forms direct contacts with RNA^27–29^ (Fig. 4i,j). It is thus possible that, in addition to enhancing export complex stability, PHAX phosphorylation also enables favorable competition with noncognate CRM1 RNA cargos.

### ARS2 is excluded from the CBC by PHAX

**Fig. 5:**
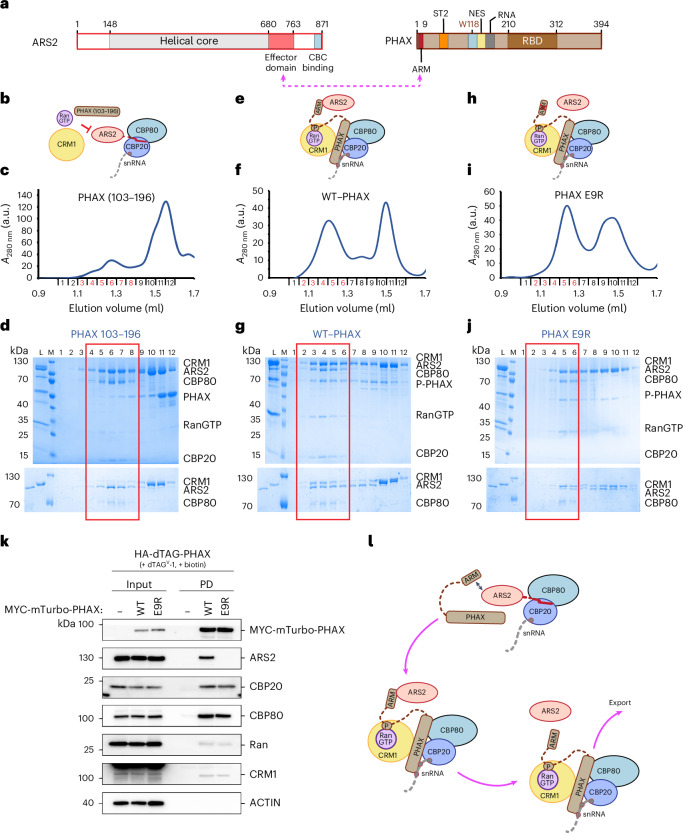
ARS2 interaction with the snRNA export complex. **a**, A schematic representation of the ARS2 and PHAX domain structure. The ARS2 effector domain binds the ARM motif in PHAX N-terminus. ARM, ARS2 recognition motif; ST2, phosphorylation site cluster 2; NES, nuclear export signal; RBD, RNA binding domain. **b**, A schematic representation of the interaction of the CBC–ARS2^147–871^–RNA complex with CRM1, RanGTP and PHAX^103–196^. **c**, The Superdex 200 gel filtration elution profile of the mixture of the CBC–ARS2^147–871^–RNA complex with CRM1, RanGTP and PHAX^103–196^. The CBC–ARS2^147–871^–RNA complex elutes in fractions highlighted in red. **d**, SDS-PAGE analysis of fractions 1–12 of the gel filtration elution profile shown in **c**. The lower panel shows a 7% SDS-PAGE analysis of the same fractions enabling better resolution of CRM1 and ARS2^147–871^. The red rectangle indicates fractions containing the CBC–ARS2^147–871^–RNA complex. CRM1, PHAX and RanGTP elute in later fractions. L, input sample loaded onto the column; M, Mw marker. **e**, A schematic representation of the interaction of the CBC–ARS2^147–871^–RNA complex with CRM1, RanGTP and FL PHAX. ARS2 binds to PHAX via its ARM motif. **f**, The Superdex 200 gel filtration elution profile of the mixture of the CBC–ARS2^147–871^–RNA complex with CRM1, RanGTP and FL PHAX. The snRNA export complex bound to ARS2^147–871^ elutes in fractions highlighted in red. **g**, SDS-PAGE analysis of fractions 1–12 of the gel filtration elution profile shown in **f**. The red rectangle indicates fractions containing the snRNA export complex bound to ARS2^147–871^. **h**, A schematic representation of the interaction of the CBC–ARS2^147–871^–RNA complex with CRM1, RanGTP and E9R mutant of FL PHAX. ARS2 binding to PHAX via its ARM motif is prevented. **i**, The Superdex 200 gel filtration elution profile of the mixture of the CBC–ARS2^147–871^–RNA complex with CRM1, RanGTP and FL PHAX E9R. **j**, SDS-PAGE analysis of fractions 1–12 of the gel filtration elution profile shown in **i**. The red rectangle indicates fractions where the snRNA export complex bound to ARS2^147–871^ eluted in **g**. Here, ARS2 becomes substoichiometric and the snRNA complex elutes later in fractions 4–6. **k**, Western blotting analysis of streptavidin pulldowns from lysates of HA-dTAG-PHAX mES cells stably expressing MYC-mTurbo tagged PHAX^WT^ and PHAX^E9R^ as described in Fig. 2j. **l**, A model for the role of ARS2 in the snRNA export complex assembly. ARS2 can simultaneously interact with the ARM motif of PHAX and bind to the groove formed at the CBP20–CBP80 interface. The latter interaction prevents PHAX binding to CBC. In the presence of CRM1–RanGTP and capped RNA, PHAX can displace ARS2 from CBC. ARS2 may remain bound to the complex via the ARM motif but might also be released, enabling its association with other CBC-effectors complexes. Source data

PHAX forms a ternary complex with CBCA^9,25^. Within this complex, the ARM of PHAX (9-EDGQL-13) binds the effector domain of ARS2^7^ (Fig. 5a), probably in a manner similar to that observed for ARS2 bound to the ARM motif of the *Schizosaccharomyces pombe* Red1 protein^13^. Consistent with this, the ARS2–PHAX interaction is lost upon mutation or deletion of the effector domain of ARS2^26,40^. In ternary complexes, ARS2 shields direct binding of PHAX, as well as the effectors NCBP3 and ZC3H18, to the CBC by sterically excluding them from the tryptophan helix binding site and the CBP20-CBP80 groove^7^. To understand how PHAX can circumvent the ARS2 block to CBC binding and to assess whether ARS2 is a component of the snRNA export complex, we first used size exclusion chromatography with purified proteins.

We assembled CBC, ARS2^147–871^ and capped RNA and incubated the complex with CRM1, RanGTP and PHAX^103–196^, which lacks the ARM motif but is able to form the snRNA complex (Fig. 2g,h). In the presence of ARS2, the snRNA export complex could no longer be formed and the CBC remained bound only to ARS2. Owing to the similar migration of CRM1 and ARS2^147–871^, this was shown by resolving the respective elution profiles on 7% sodium dodecyl sulfate polyacrylamide gel electrophoresis (SDS-PAGE) gels (Fig. 5b–d). Hence, when PHAX does not possess all its features, ARS2 blocks access to the CBC even in the presence of other snRNA export complex components. In contrast, when the CBC–ARS2^147–871^–RNA complex was incubated with CRM1, RanGTP and phosphorylated FL PHAX (which includes the ARM), the snRNA complex was formed efficiently, eluted earlier and contained ARS2^147–871^ (Fig. 5e–g). Thus, in this context, PHAX can circumvent the ARS2 block to CBC binding. We considered that ARS2 could remain connected to the complex by its effector domain binding to the ARM of PHAX or by its C-terminus binding into the CBP20–CBP80 groove^7,9^. To distinguish these possibilities, we used a FL PHAX carrying E9R mutation within the ARM, abolishing binding to ARS2^147–871^ (ref. ^7^). When the CBC–ARS2^147–871^–RNA complex was incubated with CRM1, RanGTP and phosphorylated FL PHAX E9R, the snRNA complex was formed, but the presence of ARS2^147–871^ was markedly reduced (Fig. 5h–j). Thus, without a connection between ARS2 and PHAX, the former is excluded from the export complex, probably because the reinforced binding of PHAX to the CBC in this complex outcompetes the CBC binding of ARS2.

To investigate a role of ARS2 in snRNA export complex formation in vivo, PL was performed with the PHAX E9R mutant, which revealed that the abrogated PHAX ARM-ARS2 interaction had no apparent impact on PHAX contacts with core snRNA complex subunits (Fig. 5k). This indicates that, in contrast to the NEXT complex subunit ZC3H18^7^, ARS2 is not essential for PHAX recruitment to CBC-bound RNA. While the exact role of the PHAX–ARS2 interaction remains to be elucidated, these data suggest that ARS2 may initially prevent PHAX from binding the CBC, but in the presence of CRM1–RanGTP and capped RNA, the PHAX–CBC complex can be stably formed. At this stage, ARS2 is excluded from the CBC but may remain connected via the PHAX ARM (Fig. 5l).

## Discussion

For 25 years, PHAX has been known to be crucial for snRNA export^18^, but the molecular details underlying this role have remained elusive. The structure of the snRNA export complex reported here reveals several novel aspects of PHAX function and also sheds light on the competition between RNA effectors for CBC binding.

We have recently characterized the CBCAP complex^25^ and shown that both the PHAX W118-containing helix and its C-terminus bind directly to CBC, while its N-terminal ARM motif binds to ARS2^7^. When PHAX is bound to CBC and capped RNA, only the N-terminal part of the W118-containing helix of PHAX (residues 114–132) is stably bound to CBP80^7^. However, in the presence of CRM1–RanGTP, the W118-containing helix is extended to include the PHAX NES, which binds to the canonical NES binding cleft of CRM1. Our in vitro and in vivo data demonstrate that the W118-mediated interaction of PHAX with CBC is required for snRNA export complex formation. Since CRM1 is crucial for mediating snRNA nuclear export, this interaction is probably indispensable. An equivalent CBC interaction with the W301-containing helix of ZC3H18 is similarly important for the function of the NEXT complex in RNA degradation^7^. The same surface on the CBC is also used by NCBP3, but the functional role of this interaction is not known^7^. The Trp-binding surface on the CBC is thus emerging as a key contributor to RNA fate determination through its interactions with competing effectors.

In the structure of PHAX bound to CBC and capped RNA, no contacts of PHAX with RNA were observed^7^. Within the snRNA export complex, however, PHAX strongly engages the RNA cap structure, reinforcing the binding of m^7^GTP by CBP20 and sandwiching the first transcribed nucleotide. The CBC–PHAX module thus binds tightly the entire cap structure through *π*–*π* or *π*–cation stacking interactions with the two cap nucleotides and numerous charged contacts that also include the triphosphate moiety. This mode of cap recognition is probably compatible with a Cap-1 structure bearing an additional 2′O ribose methylation of A1. Moreover, most snRNAs have an A as the first transcribed nucleotide whose further methylation to m^6^Am could possibly enhance the stacking energy between PHAX residues R147 and Y154. We further show that efficient CRM1–RanGTP recruitment requires RNA-triggered PHAX structuring, which is probably a mechanism to avoid unnecessary nuclear export of CBC and PHAX. Conversely, capped RNA is recognized by PHAX only in the presence of CRM1–RanGTP, which allows stronger binding of snRNA substrates once nuclear export is committed.

Efficient snRNA export complex formation requires PHAX phosphorylation by CK2^18,20^, but only phosphorylation of the second cluster out of eight, ST2 (residues 64–76), is necessary for export complex formation^20^. Our mass spectrometry analysis of in vitro CK2-phosphorylated PHAX revealed five phosphorylated serines in the full-length protein and four in the PHAX^41–80^ peptide, which only contains ST2. This supports that ST2 is the primary CK2 phosphorylation site in PHAX, at least in vitro. In the snRNA export complex structure, in addition to its NES binding to CRM1, PHAX also interacts with a distant region of CRM1 via its residues 55–60, containing the conserved 57-YR motif. We propose that this contact positions a ‘fuzzy’, electrostatically driven interaction between the adjacent phosphorylated ST2 cluster and the prominent basic surface of RanGTP, which further strengthens CRM1–RanGTP recruitment into the complex. Phosphorylated ST2 may also shield the basic surface on Ran from nonspecific interaction with noncognate RNA cargos. Indeed, within the snRNA export complex structure, the bulk of the RNA is not visible and PHAX specifically recognizes the cap structure. In contrast, within the tRNA, pre-miRNA and HIV RRE export complexes, the cargo RNA molecules form direct contacts with the same basic surface of Ran^27–29^.

Our structure shows that PHAX residues 112–162, the key region for snRNA export complex assembly, only fold in the simultaneous presence of CRM1–RanGTP, CBC and capped RNA. As a consequence, both CRM1–RanGTP and the capped RNA are integrated into the complex with enhanced strength. In most structurally characterized CRM1–cargo complexes, CRM1 only specifically recognizes the NES sequence. Within the snRNA export complex CRM1 is bound on multiple surfaces by the PHAX NES, PHAX^139–145^, PHAX^55–60^, ST2 and CBP20. The other well-documented case of extensive CRM1 recognition by a cargo is that of Snurportin1, which is involved in the nuclear import of snRNA^30^. However, the PHAX and Snurportin1 CRM1-binding surfaces, with the exception of their NESs, are not overlapping. The PHAX NES contains the conserved Q127 residue in the first position (Φ0), rather than a hydrophobic residue, indicative of a weak NES. In agreement, the Y154A mutant, designed not to be able to bind the RNA cap, still binds to CBC but not to CRM1–RanGTP (Fig. 3h, lanes 7–9), indicating that the PHAX NES alone does not efficiently bind CRM1.

Finally, we investigated a potential role of ARS2 within the snRNA export complex. Since ARS2 also mediates degradative pathways, we wondered whether, how and at which point ARS2 might be excluded from the committed snRNA export complex. ARS2 interacts via its effector domain with the ARM motif of PHAX and through its C-terminus with the groove at the CBP20–CBP80 interface^7,9^. We, thus, speculate that the residual association of CBC with PHAX^W108A^ (designed to prevent CBC binding) shown in Fig. 2j might be mediated by the PHAX interaction with ARS2, but this is insufficient for the recruitment of CRM1–RanGTP. In vitro, ARS2 prevents PHAX from directly binding to CBC in the CBCAP complex^7^. We now show that, in the presence of PHAX and CRM1–RanGTP, CBC interacts more efficiently with PHAX, leading to a loss of the ARS2–CBP20–CBP80 groove interaction. In this context, ARS2 probably remains only weakly linked via the PHAX ARM motif. Consequently, ARS2 might leave the complex and associate with other CBC-effector bound transcripts. In the case of ZC3H18, both the ARM-mediated interaction with ARS2 and Trp-helix binding to CBC are required for the activity of the NEXT complex^7^. The human/mouse PHAX ARM motif (9-EDGQL-13) is unique in comparison with the consensus sequence ([DE][DE]G[DE][ILV]) as it contains an unusual glutamine in the fourth position. In vitro, this exhibits lower affinity for ARS2 than ZC3H18, which has three tandem ARM motifs^7^. While our in vivo analysis confirms that the PHAX ARM mutant variant does not interact with ARS2, abolishing this interaction does not impact PHAX’s ability to recruit all snRNA export complex subunits (Fig. 5k). This suggests that, in contrast to ZC3H18, ARS2 is not strictly required for the recruitment of PHAX to CBC-bound transcripts. In the absence of a robust ARS2 interaction, PHAX possibly evolved a stronger reliance on the CBC connection.

Efficient sorting between mRNAs and short transcripts, such as snRNAs, into their respective nuclear export pathways, as well as between useful and aberrant transcripts, requires RNA effectors. PHAX is believed to be retained only on short transcripts, such as snRNAs, and is displaced from longer transcripts >200–300 nt, such as most mRNAs, by hnRNP C (refs. ^18,41^). However, the precise mechanism of the competition between PHAX and hnRNP C remains unclear. Recently, the RNA helicase UAP56/DDX39B and ALYREF, which are subunits of the transcription–export complex usually associated with mRNA export, were shown to stimulate the RNA binding of PHAX and participate in snRNA export^42^. How all these factors regulate the nuclear export of different RNAs and the exact role of the PHAX–ARS2 interaction in this mechanism remain to be further characterized.

## Methods

### Protein expression and purification

Human CBC was reconstituted as described previously^9^. Briefly, CBP80ΔNLS (lacking the N-terminal 19 residues) was expressed in High Five insect cells. Then, CBP20 was expressed in *Escherichia* *coli* BL21Star (DE3, Invitrogen) from the pETM30 vector (Gunter Stier, EMBL Heidelberg) as His-GST fusion. The cell pellets were lysed together in a buffer containing 20 mM of Tris at pH 8, 100 mM of NaCl and 3% glycerol. Lysates were clarified by centrifugation and the complex was purified on a Ni^2+^-Chelating Sepharose (Cytiva). The His-GST tag was cleaved by TEV protease. Following a subsequent passage through a Ni^2+^-chelating Sepharose, CBC was loaded on a heparin HiTrap column (Cytiva) and further purified by gel filtration on Superdex 200 Increase (Cytiva).

Human ARS2^147–871^ and FL PHAX were expressed as His-tag fusions in *E. coli* BL21Star (DE3, Invitrogen) from the pETM11 vector (Gunter Stier, EMBL Heidelberg). The proteins were purified on a Ni^2+^-chelating Sepharose (Cytiva) followed by HiTrap heparin column (Cytiva) and gel filtration on Superdex 200 Increase (Cytiva) in a buffer containing 20 mM of Tris at pH 8, 100 mM of NaCl and 3% glycerol^7^.

Human CRM1 was expressed as His-tag fusion protein from a pQE60 plasmid (Qiagen) in *E. coli* strain Tg1. Human Ran containing the Q69L mutation was expressed as His-tag fusion from a pPROEX-HTB vector (Invitrogen) in *E. coli* BL21Star (DE3, Invitrogen). Harvested cells were resuspended in 20 mM of Tris at pH 8, 150 mM of NaCl, 10 mM of MgCl_2_, 3% glycerol and 0.5 mM of TCEP, and lysed by sonication. Proteins were purified on Ni^2+^-chelating Sepharose (Cytiva) followed by Hitrap Heparin column (Cytiva). Proteins were further purified by gel filtration on Superdex 200 Increase (Cytiva) in a buffer containing 20 mM of Tris at pH 8, 100 mM of NaCl and 2% glycerol.

PHAX^103–196^ and its mutant variants domains were expressed in *E. coli* BL21Star (DE3, Invitrogen) from the pETM41 vector (Gunter Stier, EMBL Heidelberg) as His-MBP fusion. The proteins were purified on a Ni^2+^-chelating Sepharose (Cytiva) followed by gel filtration on Superdex 200 Increase (Cytiva) in a buffer containing 20 mM of Tris at pH 8, 100 mM of NaCl and 2% glycerol^7^.

### RNA preparation

U1SnRNA was transcribed in a 250 μl volume containing 12.5 μg of T7 RNA Polymerase (Promega), 10 mM of DTT, 10 U RNasin Plus (Promega), NTP mixture (1 mM of ATP, 1 mM of CTP, 1 mM of UTP, 1 mM of GTP and 4 mM of Anti-Reverse Cap Analog (ARCA) (NEB)), 3 μg of DNA template, 1 mM of spermidine, 15 mM of MgCl_2_ and 50 mM of Tris pH 8, for 3 h at 37 °C. The sample was treated with 40 U of DNAse I for 10 min at 37 °C. RNA was purified using Zymo R1017 columns following the manufacturer’s instructions. The transcription product was analyzed on SDS-PAGE. RNA was refolded at 95 °C for 5 min followed by cooling at room temperature for 1 h in the presence of 1 mM of MgCl_2._

### Protein phosphorylation

Then, 100 μg of PHAX purified protein was incubated for 1 h at room temperature in presence of 1,000 U of CK2, 100 μM of ATP and 2.5 μl of reaction buffer (NEB).

### RanGTP/GDP exchange

Typically, 30 nmol of Ran were incubated in a total volume of 100 μl with 10 mM of EDTA and 7% phosphatase alkaline at room temperature for 2 h. The sample was spun at 2,000*g* for 10 min and purified on Superdex 200 Increase (Cytiva) in a buffer containing 20 mM of Tris at pH 8, 100 mM of NaCl and 2% glycerol. The protein sample was mixed with 1 mM of GTP and purified on Superdex 200 Increase (Cytiva) in a buffer containing 20 mM of Tris at pH 8, 100 mM of NaCl, 2% glycerol and 50 μM of GTP, flash frozen in liquid nitrogen and stored at −80 °C.

### Assembly of the snRNA export complex

Next, 4 nmol of CBC and 8 nmol of phosphorylated PHAX, CRM1 and RanGTP were mixed to a total volume of 50 μl in the presence of 100 μM of U1 snRNA or short capped RNA (m^7^GpppAAUCUAUAAUAGCA), 1 mM of Mg^2+^, 1 mM of KCl and 100 μM of GTP. The complex was loaded on Superdex 200 Increase 3.2/300 with 100 mM of NaCl, 20 mM of Tris at pH 8, 1 mM of Mg^2+^, 0.5 mM of TCEP, 2% glycerol and 10 μM of GTP. Purified sample was used directly for cryo-EM grid preparation.

### Complex assembly analysis by gel filtration

Then, 180 pmol of CBC and 360 pmol of the different PHAX constructs, CRM1 and RanGTP were mixed in a total volume of 50 μl in the presence of 100 μM of capped RNA (m^7^GpppAAUCUAUAAUAGCA), cap-analog m^7^GpppG or m^7^GTP and 1 mM of Mg^2+^, 1 mM of KCl and 100 μM of GTP. Samples were incubated for 30 min on ice before analysis on Superdex 200 Increase 3.2/300. Next, 50 μl fractions were collected and analyzed on SDS-PAGE.

### ITC

ITC experiments were performed at 25 °C using a PEAQ-ITC micro-calorimeter (MicroCal). Experiments included one 0.5 µl injection and 15–18 injections of 1.5 µl at 0.2–0.25 mM of m^7^GTP, m^7^GpppG or PHAX^103–196^ and its R147E, Y152E or Y154E mutant into the sample cell that contained 20 µM of CBC in the presence or absence of 0.1 mM of m^7^GTP or m^7^GpppG, in 20 mM of Tris at pH 8.0, 100 mM of NaCl and 2% glycerol. The initial data point was deleted from the data sets. Binding isotherms were fitted with a one-site binding model by nonlinear regression using the MicroCal PEAQ-ITC Analysis software. Measurements were performed in duplicate.

### Mass photometry analysis

Mass photometry measurements were performed on a OneMP mass photometer (Refeyn). Coverslips (no. 1.5H, 24 × 50 mm, VWR) were washed with water and isopropanol before being used as a support for silicone gaskets (CultureWell 423 Reusable Gaskets, Grace Bio-labs). Contrast/mass calibration was realized using native marker (Native Marker unstained protein 426 standard, LC0725, Life Technologies) with a medium field of view and monitored for 60 s using the AcquireMP software (Refeyn). For each condition, 18 µl of buffer 100 mM of NaCl, 20 mM of NaCl, 1 mM of Mg^2+^, 0.5 mM of TCEP, 2% glycerol and 10 μM of GTP were used to find the focus. Next, 2 µl of diluted purified complex or diluted equimolar mix of the different proteins and RNA (m^7^GpppAAUCUAUAAUAGCA) (200 nM) were added to reach a final concentration of 20 nM. Movies of 60 s duration were recorded using AcquireMP software (v2024 R1, Refeyn). The data were analyzed and mass estimated automatically using the DiscoverMP software (v2024 R1, Refeyn).

### Cell culture and transfections

All mES cell lines used or generated in this study were descendants of wildtype ES-E14TG2a cells (male genotype, XY). mES cells were cultured on 0.2% gelatin coated plates in 2i/LIF (leukemia inhibitory factor) containing media (1:1 mix of DMEM/F12 (Thermo Fisher) and Neurobasal (Thermo Fisher) supplemented with 1% (v/v) penicillin–streptomycin (Sigma), 2 µM of Glutamax (Thermo Fisher), 0.1 mM of nonessential amino acids (Thermo Fisher), 1 mM of sodium pyruvate (Thermo Fisher), 0.5x N-2 Supplement (Thermo Fisher), 0.5x B-27 Supplement (Thermo Fisher), 50 µM of 2-mercaptoethanol (Thermo Fisher), 3 µM of GSK3-inhibitor (CHIR99021, Sigma), 1 µM of MEK-inhibitor (PD0325901, Sigma) and LIF (produced in house)) at 37 °C and 5% CO_2_. Cells were passaged every 48–72 h by dissociating cells with 0.05% trypsin (Sigma) before neutralizing with an equal volume of 1x trypsin inhibitor (Thermo Fisher). Cells were pelleted by centrifugation to remove Trypsin before resuspending in 2i/LIF media and plating at ~ 8 × 10^4^ cells ml^−1^.

The generation of CRISPR/Cas9-mediated genomic knock-ins of N-terminal 2xHA-FKBP-V(HA-dTAG)-PHAX mES cells was carried out as described in ref. ^45^. Depletion of HA-dTAG-PHAX was performed by the addition of dTAG^V^-1 (Tocris) to the cell culture medium to a final concentration of 100 nM.

### cDNA cloning and exogenous expression of PHAX

Full length mouse PHAX cDNA was cloned from cDNA library synthesis from 2 µg of total mouse RNA using Superscript III reverse transcription reagents (Thermo Fisher) into a pCR8/GW/TOPO Gateway entry plasmid (Thermo Fisher) by TA TOPO cloning. PHAX mutations were introduced into pCR8[mouse PHAX] plasmids by polymerase chain reaction. These were then used as templates to introduce into a piggyBAC (pB) vector containing an N-terminal MYC and miniTurbo (MYC-mTurbo) tag using NEBbuilder HiFi DNA assembly (NEB). Wildtype mES cells were transfected with pB[MYC-] or pB[MYC-mTurbo-PHAX] vectors along with a pB transposase expressing vector (pBase) in a 1:1 ratio using Viafect (Promega). Cell pools were selected for successful integration of pB plasmids with Blastocidin (BSD) for 7–10 days until negative control cells no longer survived. The expression of constructs were validated by western blotting analysis using MYC antibodies.

### In vivo PL and streptavidin enrichment

In vivo PL with MYC-mTurbo-PHAX variants was stimulated by supplementing the media with 50 µM of biotin for 4 h. Cells expressing MYC without the mTurbo fusion were used in parallel as negative controls. For the enrichment of biotinylated proteins, cells were lysed in HT150 extraction buffer (20 mM of HEPES at pH 7.4, 150 mM of NaCl and 0.5% v/v Triton X-100) freshly supplemented with protease inhibitors. Lysates were sheared by sonication (3 × 5 s, amplitude 2) and cleared by centrifugation at 18,000*g* for 20 min. Biotinylated proteins were enriched by incubating with Dynabeads MyOne Streptavidin C1 beads (ThermoFisher) overnight at 4 °C. Beads were washed three times with HT150 extraction buffer, transferring beads to a fresh tube on the final wash. Proteins were eluted by boiling in 1X NuPAGE loading buffer (Invitrogen) for 5 min. Supernatants were mixed with 10X Reducing Agent (Invitrogen) and denatured for a further 5 min at 95 °C before proceeding with western blotting analysis. To assess biotinylation, membranes were first blocked in 10% w/v BSA before probing with horseradish peroxidase-conjugated streptavidin (ThermoFisher). The bands were visualized by SuperSignal West Femto chemiluminescent ECL (Thermo) and captured using the Amersham Imager 600 or ImageQuant 800 imagine systems (GE Healthcare).

### Cryo-EM sample preparation

EM grids were glow-discharged on each side for 90 s at 30 standard cubic centimeters, with 100% power with a mixture of 90% argon and 10% oxygen using the Fischione 1070 Nanoclean plasma cleaner. Then, 3.5 μl of the sample was applied to 300 mesh Quantifoil R 1.2/1.3 glow-discharged grids, at 4 °C, 100% humidity and blotting for 2 s at a blot force of −2 in a Vitrobot Mark IV.

### Cryo-EM data collection and processing

Grids were initially screened using a Glacios Cryo-TEM equipped with a Falcon 4i direct electron detector and SelectrisX energy filter (Thermo Fisher Scientific). Final data collection was performed on a Titan Krios TEM (Thermo Fisher Scientific) operated at 300 kV, equipped with a Gatan K3 and a GIF Quantum energy filter (Gatan). In total, 8,000 movies were collected at 105k magnification giving a pixel size of 0.84 Å, a defocus range from −1.0 to −2.2 µm in 0.2 µm steps and a total dose of ~40 e^−^ Å^−2^ per movie and 40 frames per movie. The cryo-EM data collection was performed using the EPU 3 software (Thermo Fisher Scientific).

Data processing was performed using CryoSPARC v4.3 (ref. ^46^). After movie drift correction and CTF parameter determination, realigned micrographs were manually inspected and 7,302 micrographs were selected. Particles were initially selected from 665 representative micrographs using Blob Picker with a diameter ranging from 110 Å to 190 Å, and then extracted with a box size of 400 × 400 Å^2^. The best particles were used to generate a Topaz model or to create templates for particle selection. After extraction, duplicates were removed and two-dimensional classification was applied to eliminate particles displaying poor structural features and to select two-dimensional class averages with lower background noise. ‘Ab-initio’ reconstruction was performed, followed by ‘heterogeneous refinement’ and ‘three-dimensional classification’ to further select particles. ‘Nonuniform refinement’ was performed on the final sets of particles, followed by ‘reference based motion correction’ and a final ‘nonuniform refinement’. The final map was at an average resolution of 2.62 Å (FSC 0.143 threshold) (Table 1). On the basis of this consensus map, particle subtraction around CBC and CRM1/RanGTP was performed. The subtracted particles were finally subject to local refinement to improve subtracted particle angles and shift estimation. All final maps were used to calculate directional FSC and local resolution in CryoSPARC. The detailed processing workflow, including data statistics, is shown in Extended Data Fig. 2.

### Model building and refinement

Model building, using available structures of cap-bound CBC and CRM1–RanGTP as a guide, was initially performed in COOT^47^ using the high-resolution maps focused on the CBC or CRM1–RanGTP moieties of the snRNA export complex. The AF2 prediction of the complete complex was also useful. Since PHAX is at the slightly flexible interface between the two lobes of the complex, final refinement of the complete complex was against the composite focused map. Models were refined using Phenix real-space refinement^48^ with Ramachandran restraints (Table 1). Structure figures were prepared using ChimeraX^49^ and Pymol^44^.

### Statistics and reproducibility

The gel filtration experiments in Figs. 1d,f,g, 2h,i, 3h and 5d,g,j were performed twice. The gel filtration experiment in Extended Data Fig. 2b is a duplicate of Fig. 1f at a higher protein concentration. The gel filtration experiments in Extended Data Fig. 5b,e were performed twice. The streptavidin pulldowns in Figs. 2j, 3i, 4g and 5k were carried out twice. The western blot analysis of lysates from WT and HA-dTAG-PHAX mES cells shown in Extended Data Fig. 3d were performed twice. The western blot analysis of lysates from WT control or HA-dTAG-PHAX mES cells expressing MYC-miniTurbo(mT)-PHAX proteins shown in Extended Data Fig. 3e were performed twice.

### Reporting summary

Further information on research design is available in the Nature Portfolio Reporting Summary linked to this article.

## Online content

Any methods, additional references, Nature Portfolio reporting summaries, source data, extended data, supplementary information, acknowledgments, peer review information; details of author contributions and competing interests; and statements of data and code availability are available at 10.1038/s41594-025-01595-5.

## Supplementary information

Supplementary Information Supplementary Fig. 1.

Reporting Summary

## Source data

Source Data Fig. 1 Unprocessed gels.

Source Data Fig. 2 Unprocessed gels and western blots.

Source Data Fig. 3 Unprocessed gels and western blots.

Source Data Fig. 4 Unprocessed western blots.

Source Data Fig. 5 Unprocessed gels and western blots.

Source Data Extended Data Fig. 2 Unprocessed gels.

Source Data Extended Data Fig. 3 Unprocessed western blots.

Source Data Extended Data Fig. 5 Unprocessed gels.

## Data Availability

The atomic coordinates and cryo-EM maps of the human snRNA export complex determined in this study have been deposited under the PDB accession code 9HFL and EMDB code 52115. Source data are provided with this paper.
