# Supplementary material for: Structural basis for the synergistic assembly of the snRNA export complex
Source: Nat Struct Mol Biol. 2025 Jul 3;32(8):1555–66. doi: 10.1038/s41594-025-01595-5 (PMC12350170; doi:10.1038/s41594-025-01595-5)
Supplement: Supplementary file 1 — Supplementary Fig. 1. [file 41594_2025_1595_MOESM1_ESM.pdf]

nature structural & molecular biology

Supplementary information https://doi.org/10.1038/s41594-025-01595-5

# Structural basis for the synergistic assembly of the snRNA export complex

In the format provided by the authors and unedited

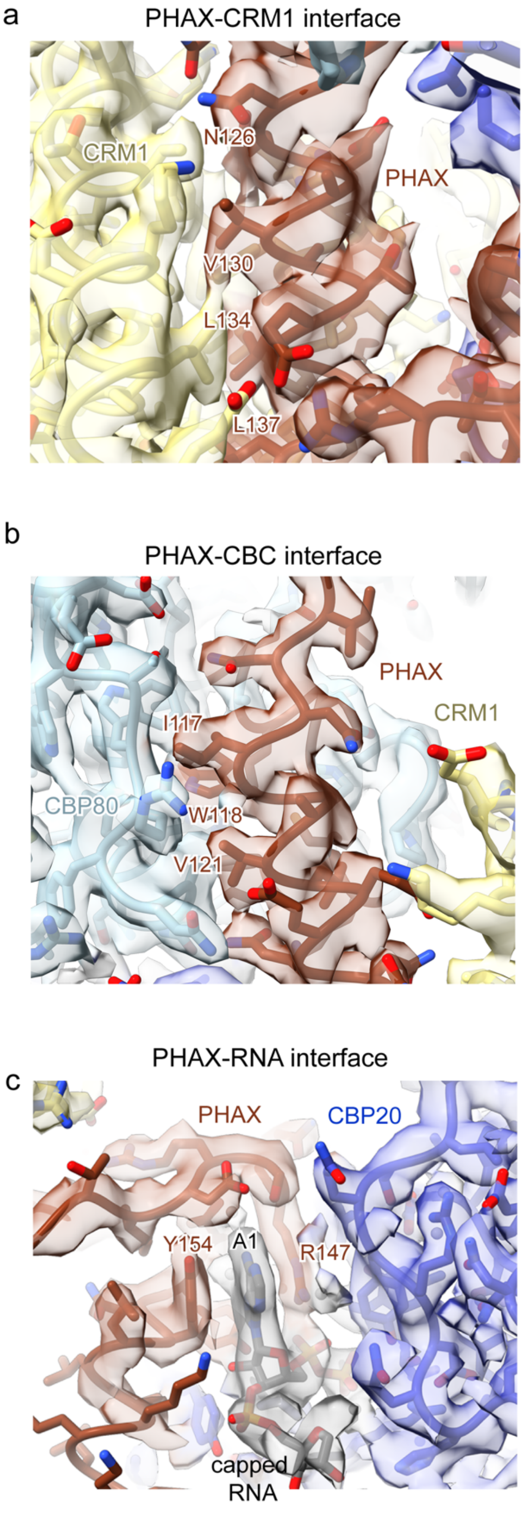


**Supplementary Figure 1. Cryo-EM density of the snRNA export complex.**
**a.** Cryo-EM density of the PHAX-CRM1 interface**. b.** Cryo-EM density of the PHAX-CBC interface**. c.** Cryo-EM density of the PHAX-RNA interface**.**
