# Supplementary material for: Structural basis for the synergistic assembly of the snRNA export complex
Source: Nat Struct Mol Biol. 2025 Jul 3;32(8):1555–66. doi: 10.1038/s41594-025-01595-5 (PMC12350170; doi:10.1038/s41594-025-01595-5)
Supplement: Supplementary file 2 — Reporting Summary [file 41594_2025_1595_MOESM2_ESM.pdf]

nature portfolio

Corresponding author(s): Jan KADLEC and Stephen CUSACK

Last updated by author(s): 2025 04 16

Reporting Summary

Nature Portfolio wishes to improve the reproducibility of the work that we publish. This form provides structure for consistency and transparency in reporting. For further information on Nature Portfolio policies, see our Editorial Policies and the Editorial Policy Checklist.

Statistics

For all statistical analyses, confirm that the following items are present in the figure legend, table legend, main text, or Methods section.

n/a	Confirmed	
☒	☐	The exact sample size (*n*) for each experimental group/condition, given as a discrete number and unit of measurement
☐	☒	A statement on whether measurements were taken from distinct samples or whether the same sample was measured repeatedly
☒	☐	The statistical test(s) used AND whether they are one- or two-sided<br>*Only common tests should be described solely by name; describe more complex techniques in the Methods section.*
☒	☐	A description of all covariates tested
☒	☐	A description of any assumptions or corrections, such as tests of normality and adjustment for multiple comparisons
☒	☐	A full description of the statistical parameters including central tendency (e.g. means) or other basic estimates (e.g. regression coefficient) AND variation (e.g. standard deviation) or associated estimates of uncertainty (e.g. confidence intervals)
☒	☐	For null hypothesis testing, the test statistic (e.g. *F*, *t*, *r*) with confidence intervals, effect sizes, degrees of freedom and *P* value noted<br>*Give P values as exact values whenever suitable.*
☒	☐	For Bayesian analysis, information on the choice of priors and Markov chain Monte Carlo settings
☒	☐	For hierarchical and complex designs, identification of the appropriate level for tests and full reporting of outcomes
☒	☐	Estimates of effect sizes (e.g. Cohen's *d*, Pearson's *r*), indicating how they were calculated

Our web collection on statistics for biologists contains articles on many of the points above.

Software and code

Policy information about availability of computer code

Data collection	The cryo-EM data collection was performed using the EPU 3 software (Thermo Fisher Scientific). LC-ESI-MS data were collected using Mass hunter (v. 11.0 Agilent Technologies). MALDI-MS data were collected using flexControl™ (v.3.4, Bruker Daltonik). Mass Photometry data were recorded using AcquireMP software (v2024 R1, Refeyn).
Data analysis	All the software used for cryo-EM data analysis and structure modeling is publicly available and fully referenced in the manuscript. ITC data was analyzed using the MicroCal PEAQ-ITC Analysis software. Mass Photometry data analysed and mass estimated automatically using the DiscoverMP software (v2024 R1, Refeyn). LC-ESI-MS data were processed using Bioconfirm (v. 12.01, Agilent Technologies) and GPMAW (v. 7.00b2, Lighthouse Data, Denmark) software. MALDI-MS were processed using flexAnalysis™ (v. 3.4, Bruker Daltonik).

For manuscripts utilizing custom algorithms or software that are central to the research but not yet described in published literature, software must be made available to editors and reviewers. We strongly encourage code deposition in a community repository (e.g. GitHub). See the Nature Portfolio guidelines for submitting code & software for further information.

Data

Policy information about availability of data

All manuscripts must include a data availability statement. This statement should provide the following information, where applicable:

- Accession codes, unique identifiers, or web links for publicly available datasets
- A description of any restrictions on data availability
- For clinical datasets or third party data, please ensure that the statement adheres to our policy

The atomic coordinates and cryo-EM maps of the human snRNA export complex determined in this study have been deposited under the PDB accession code 9HFL and EMDB code 52115, respectively.

Research involving human participants, their data, or biological material

Policy information about studies with human participants or human data. See also policy information about sex, gender (identity/presentation), and sexual orientation and race, ethnicity and racism.

Reporting on sex and gender	No human participants, data and biological materials are involved in this study.
Reporting on race, ethnicity, or other socially relevant groupings	No human participants, data and biological materials are involved in this study.
Population characteristics	No human participants, data and biological materials are involved in this study.
Recruitment	No human participants, data and biological materials are involved in this study.
Ethics oversight	No human participants, data and biological materials are involved in this study.

Note that full information on the approval of the study protocol must also be provided in the manuscript.

Field-specific reporting

Please select the one below that is the best fit for your research. If you are not sure, read the appropriate sections before making your selection.

☒ Life sciences ☐ Behavioural & social sciences ☐ Ecological, evolutionary & environmental sciences

For a reference copy of the document with all sections, see nature.com/documents/nr-reporting-summary-flat.pdf

Life sciences study design

All studies must disclose on these points even when the disclosure is negative.

Sample size	No sample size calculation was performed in this study. The study does not include any statistics. Only structural analysis and protein interaction studies.
Data exclusions	No data was excluded from the study
Replication	Number of replicates was minimum 2. All attempts at replication were successful.
Randomization	No experimental groups involved in this study, thus randomization was not performed.
Blinding	No experiments required bliding in this study. The study does not include any statistics

Reporting for specific materials, systems and methods

We require information from authors about some types of materials, experimental systems and methods used in many studies. Here, indicate whether each material, system or method listed is relevant to your study. If you are not sure if a list item applies to your research, read the appropriate section before selecting a response.

Materials & experimental systems

n/a	Involved in the study	
☐	☒	Antibodies
☐	☒	Eukaryotic cell lines
☒	☐	Palaeontology and archaeology
☒	☐	Animals and other organisms
☒	☐	Clinical data
☒	☐	Dual use research of concern
☒	☐	Plants

Methods

n/a	Involved in the study	
☒	☐	ChIP-seq
☒	☐	Flow cytometry
☒	☐	MRI-based neuroimaging

Antibodies

Antibodies used

MYC (Cell Signalling Cat#2278, RRID:AB_490778), Ran (Proteintech Cat#10469-1AP, RRID:AB_2176484), ARS2 (Genetex Cat#GTX119872, AB_10720168), ACTIN (Sigma-Aldrich Cat# A2228, RRID:AB_476697), HA (Roche Cat# 11867423001, RRID:AB_390918), CRM1 (Proteintech Cat# 66763-1-Ig, RRID:AB_2882109), PHAX (Proteintech Cat# 16481-1-AP, RRID:AB_2299663), CBP20 and CBP80 antibodies were a kind gift from E. Izaurralde (generated in DOI:10.1016/0092-8674(94)90530-4)

Validation

Antibodies were validated by western blotting.
Antibodies were also independently validated by the manufacturers or sources:
MYC: https://www.cellsignal.com/products/primary-antibodies/myc-tag-71d10-rabbit-mab/2278
Ran: https://www.ptglab.com/products/RAN-Antibody-10469-1-AP.htm
ARS2: https://www.genetex.com/Product/Detail/ARS2-antibody/GTX119872
ACTIN: https://www.sigmaaldrich.com/DK/en/product/sigma/a2228
HA: https://www.sigmaaldrich.com/DK/en/product/roche/roahaha
CRM1: https://www.ptglab.com/products/XPO1-Antibody-66763-1-Ig.htm
PHAX: https://www.ptglab.com/products/PHAX-Antibody-16481-1-AP.htm
CBP20: DOI:10.1016/0092-8674(94)90530-4
CBP80:DOI:10.1016/0092-8674(94)90530-4

Eukaryotic cell lines

Policy information about cell lines and Sex and Gender in Research

Cell line source(s)

All mouse embryonic stem (mES) cell lines used or generated in this study were descendants of wild type ES-E14TG2a cells (male genotype, XY, ATCC:#CRL-1821, RRID:CVCL_9108). High Five insect cells (BTI-TN-5B1-4, Thermo Fisher, Cat #B85502).

Authentication

CRISPR knock-in cell lines were validated by genotyping of the edited locus and western blot analysis for tagged proteins. High Five insect cells were not authenticated.

Mycoplasma contamination

Parental cell lines were regularly tested for mycoplasma contamination using Eurofins and tested negative. High Five insect cells were not tested.

Commonly misidentified lines
(See ICLAC register)

No commonly misidentified cell lines were used in this study.

Plants

Seed stocks

Report on the source of all seed stocks or other plant material used. If applicable, state the seed stock centre and catalogue number. If plant specimens were collected from the field, describe the collection location, date and sampling procedures.

Novel plant genotypes

Describe the methods by which all novel plant genotypes were produced. This includes those generated by transgenic approaches, gene editing, chemical/radiation-based mutagenesis and hybridization. For transgenic lines, describe the transformation method, the number of independent lines analyzed and the generation upon which experiments were performed. For gene-edited lines, describe the editor used, the endogenous sequence targeted for editing, the targeting guide RNA sequence (if applicable) and how the editor was applied.

Authentication

Describe any authentication procedures for each seed stock used or novel genotype generated. Describe any experiments used to assess the effect of a mutation and, where applicable, how potential secondary effects (e.g. second site T-DNA insertions, mosiacism, off-target gene editing) were examined.
