# Supplementary figures and images for: Structural basis for the synergistic assembly of the snRNA export complex
Source: Nat Struct Mol Biol. 2025 Jul 3;32(8):1555–66. doi: 10.1038/s41594-025-01595-5 (PMC12350170; doi:10.1038/s41594-025-01595-5)

Figure 1d

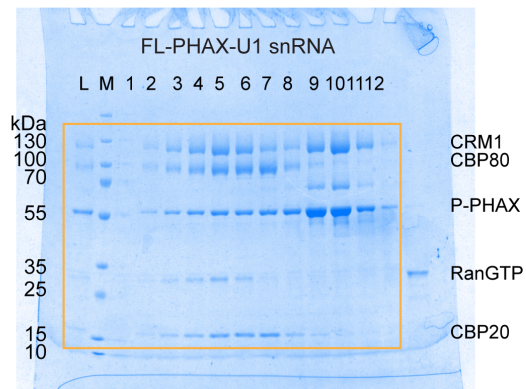

Figure 1f

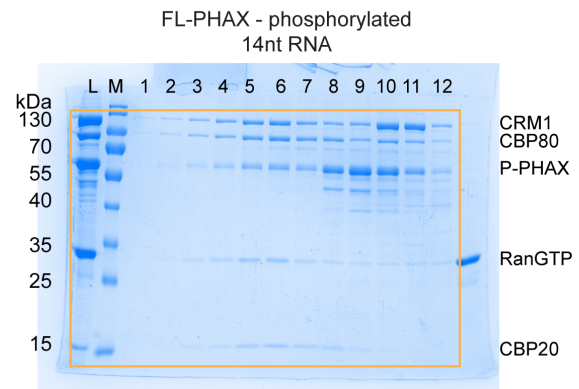

Figure 1g

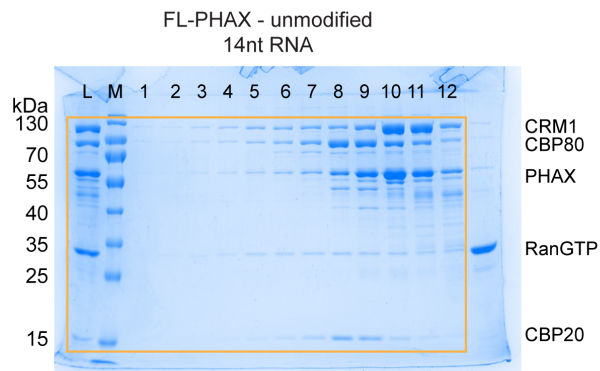

Supplement: Supplementary file 3 — Unprocessed gels. [file 41594_2025_1595_MOESM3_ESM.pdf]

Figure 2h

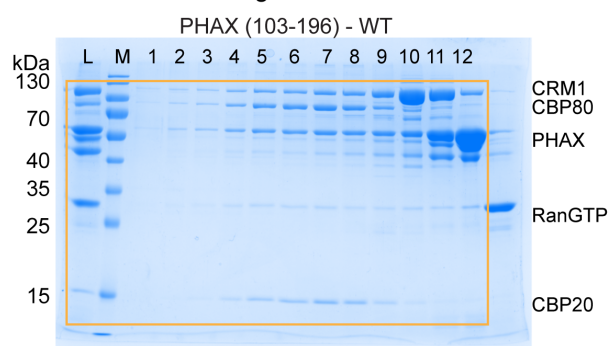

Figure 2i

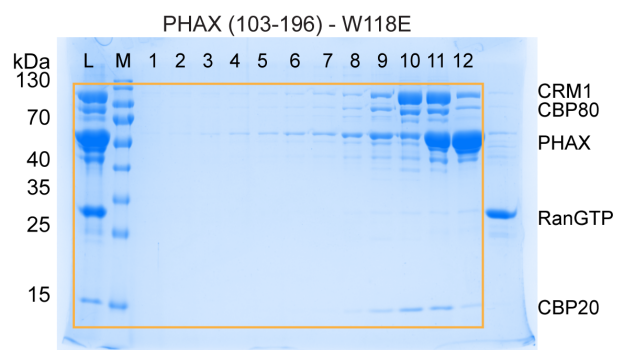

Figure 2j

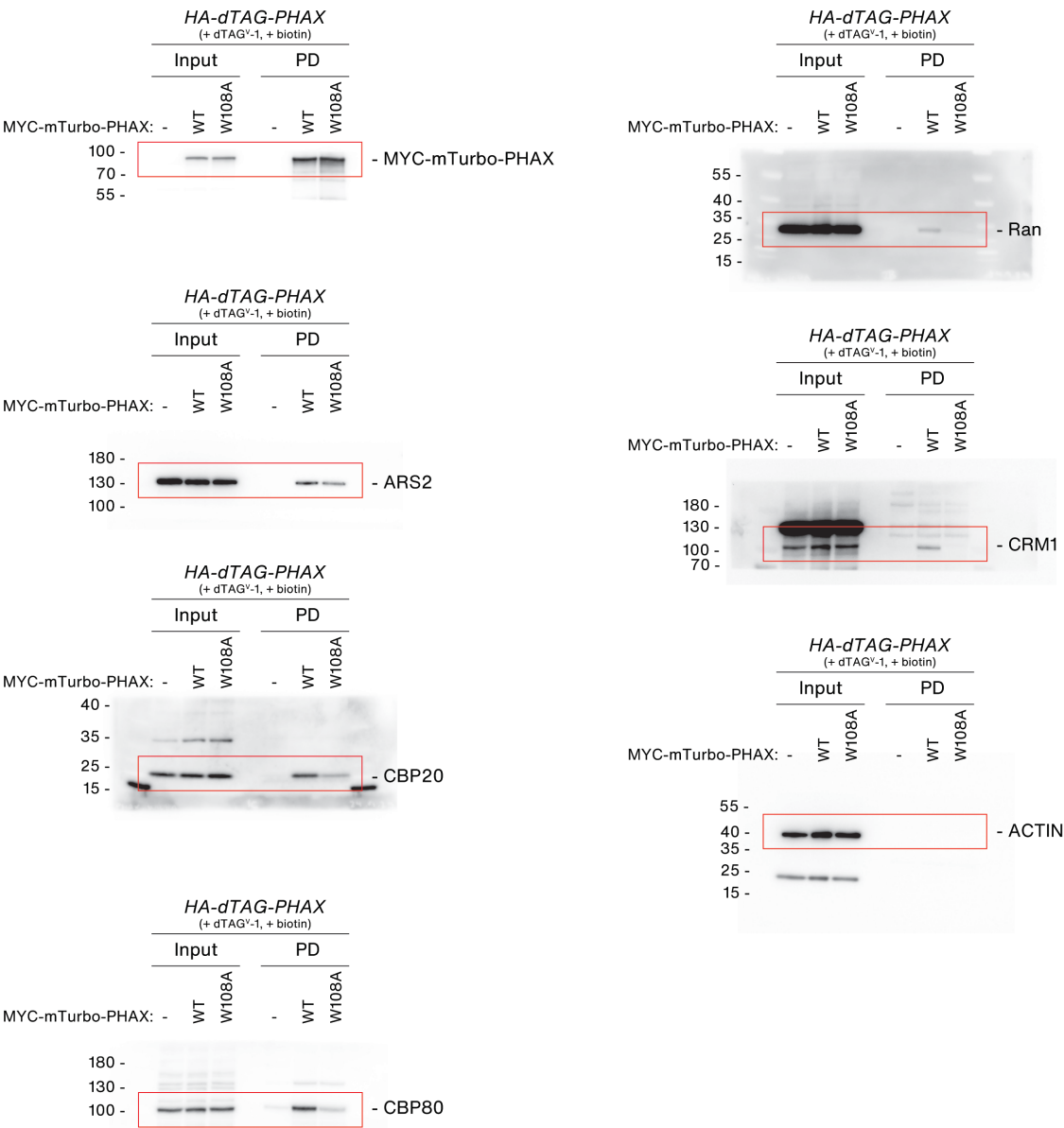

Supplement: Supplementary file 4 — Unprocessed gels and western blots. [file 41594_2025_1595_MOESM4_ESM.pdf]

Figure 3h

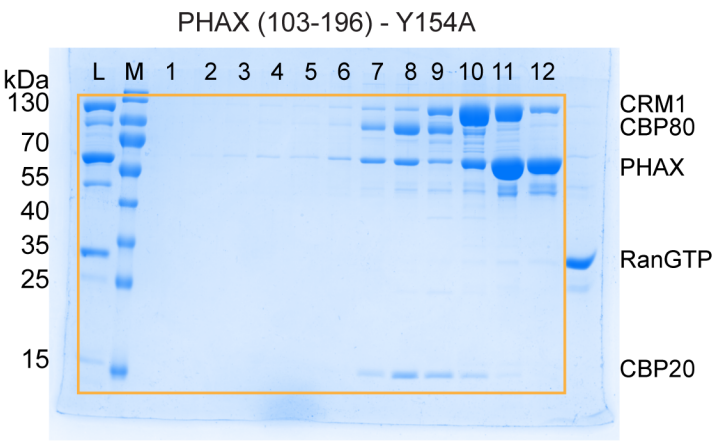

Figure 3i

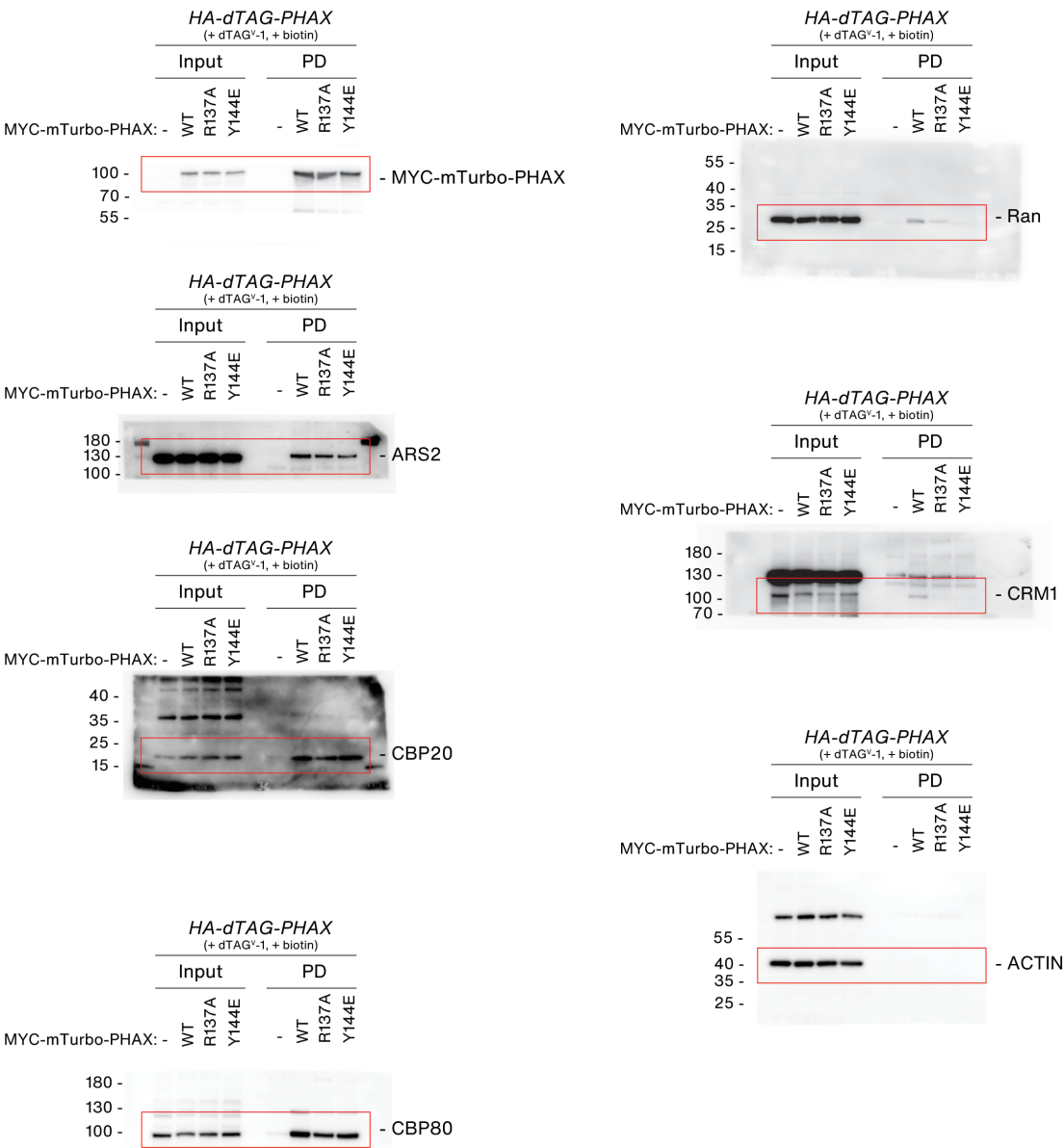

Supplement: Supplementary file 5 — Unprocessed gels and western blots. [file 41594_2025_1595_MOESM5_ESM.pdf]

Figure 4g

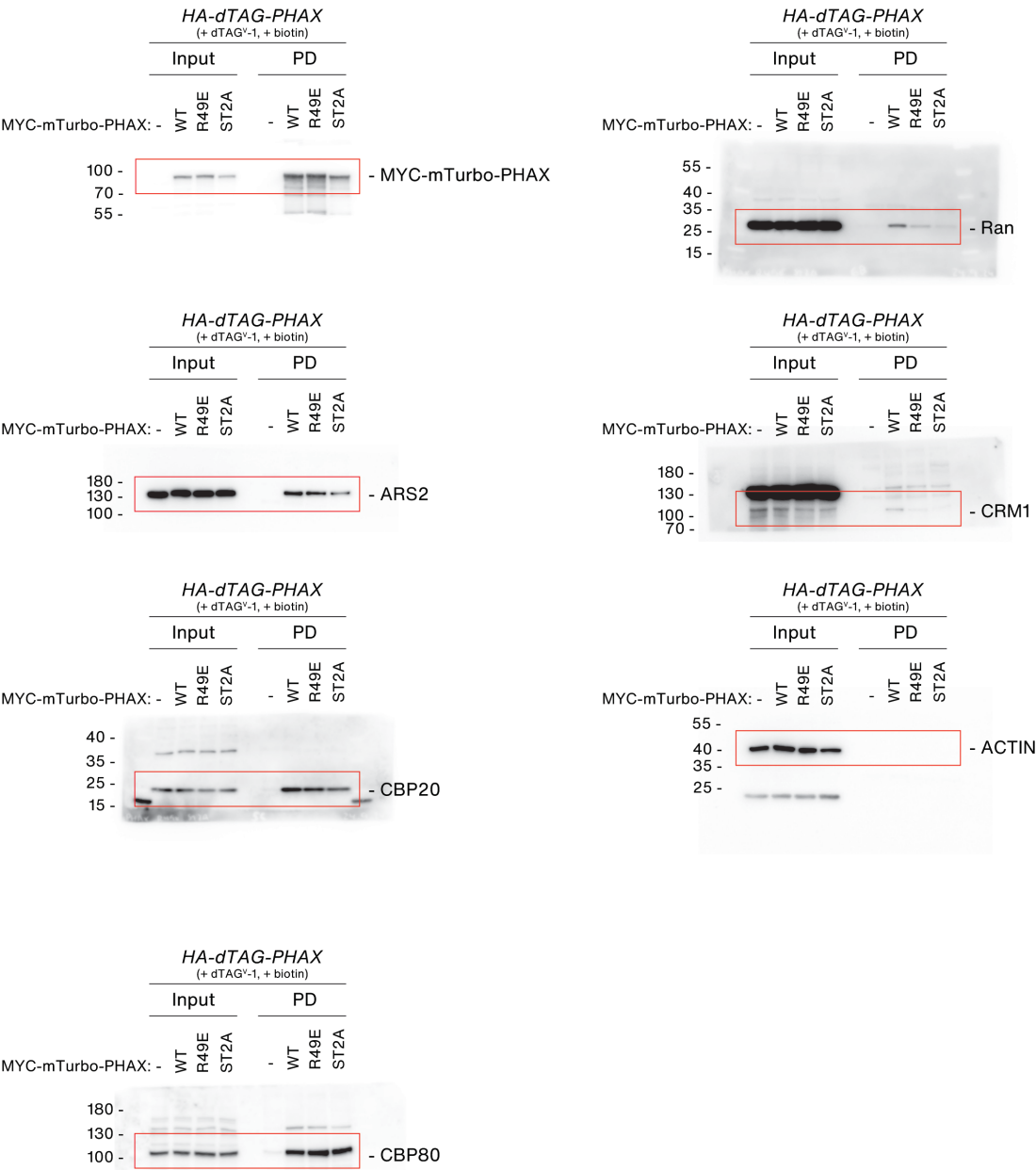

Supplement: Supplementary file 6 — Unprocessed western blots. [file 41594_2025_1595_MOESM6_ESM.pdf]

Figure 5d

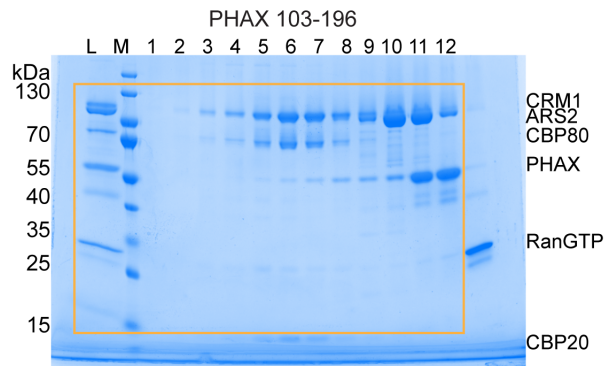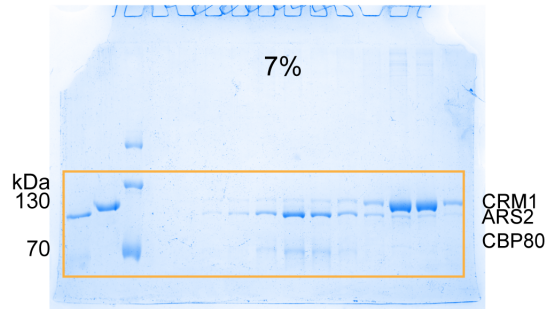

Figure 5g

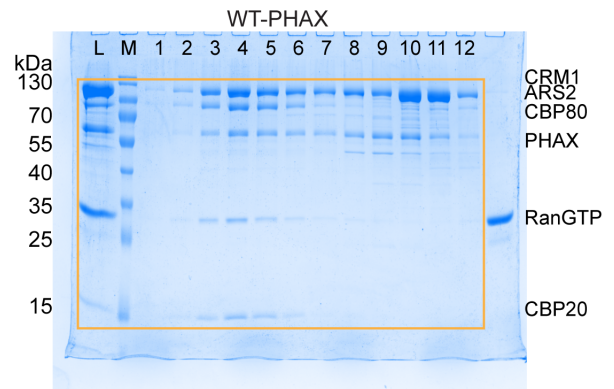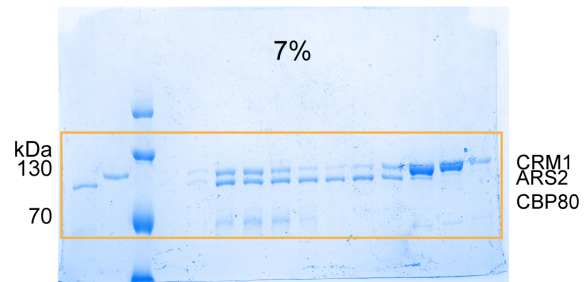

Figure 5j

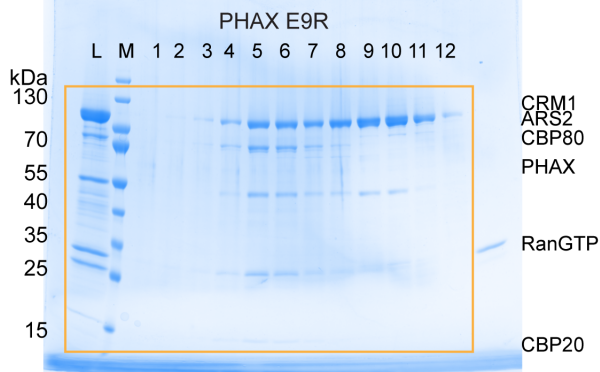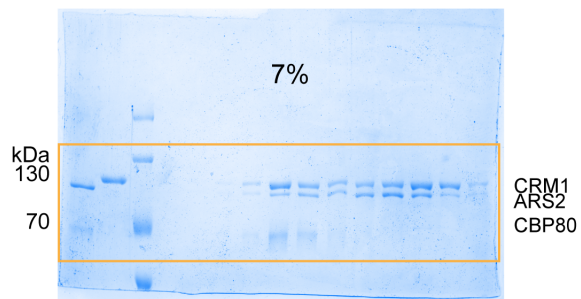

Figure 5k

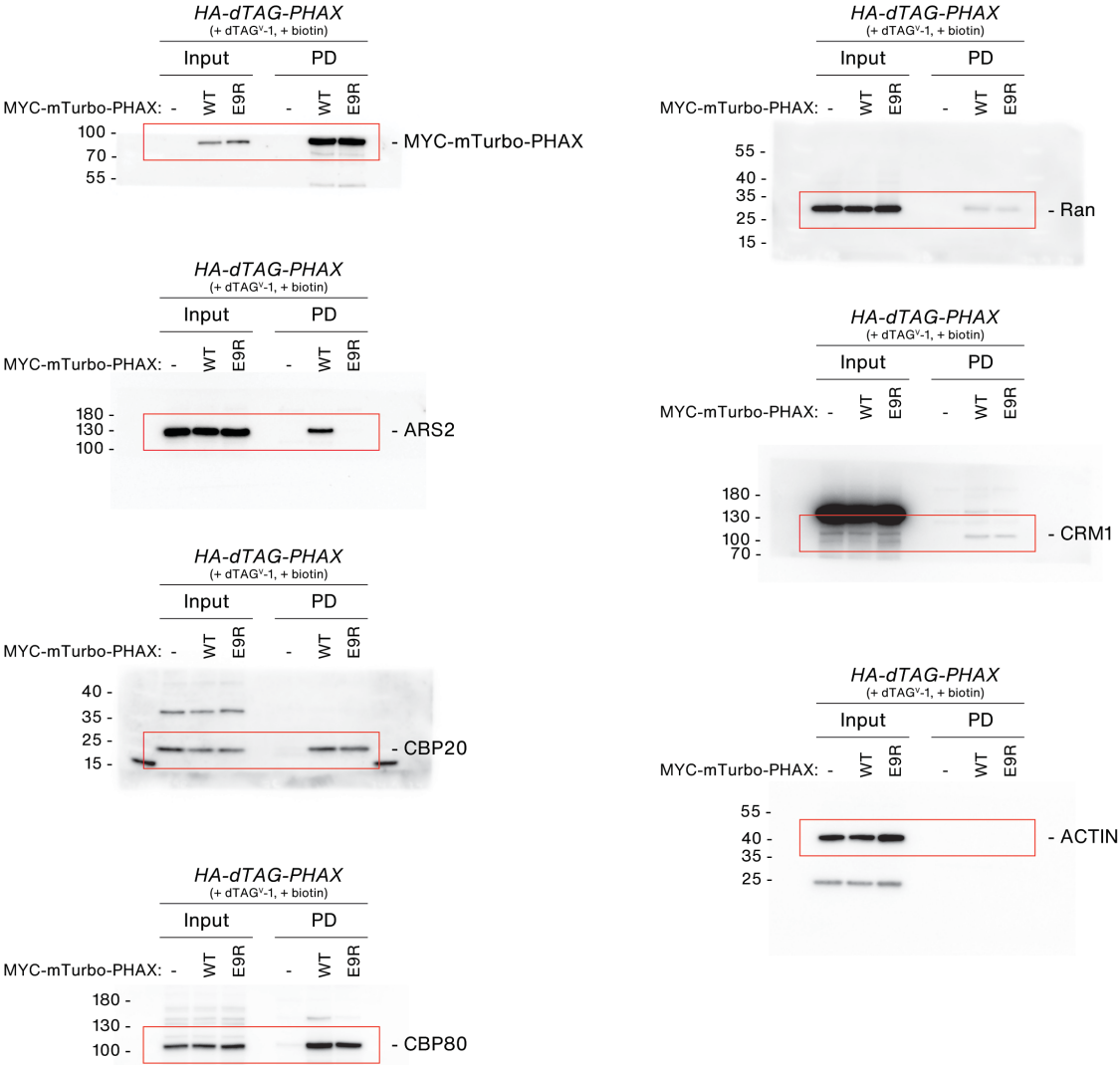

Supplement: Supplementary file 7 — Unprocessed gels and western blots. [file 41594_2025_1595_MOESM7_ESM.pdf]

# Extended data Figure 2e

FL-PHAX - phosphorylated  
14nt RNA

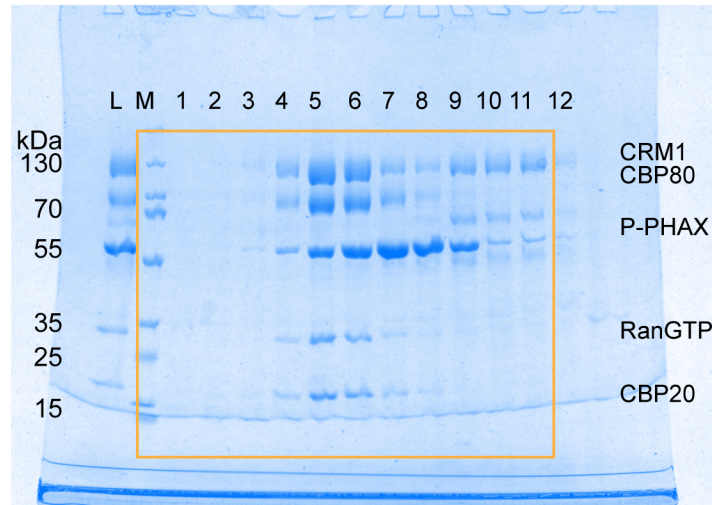

Supplement: Supplementary file 8 — Unprocessed gels. [file 41594_2025_1595_MOESM8_ESM.pdf]

# Extended data Figure 3d

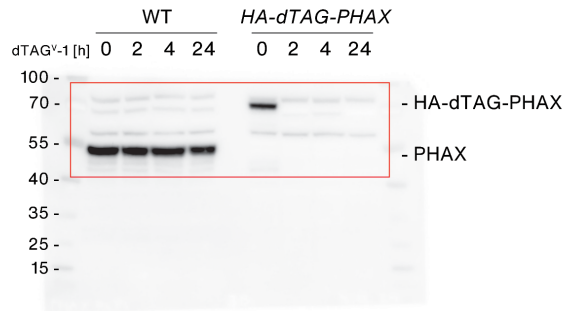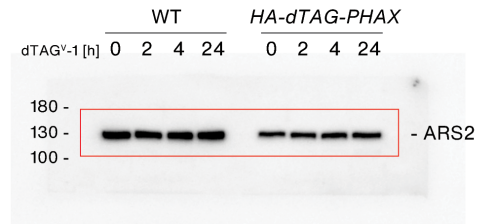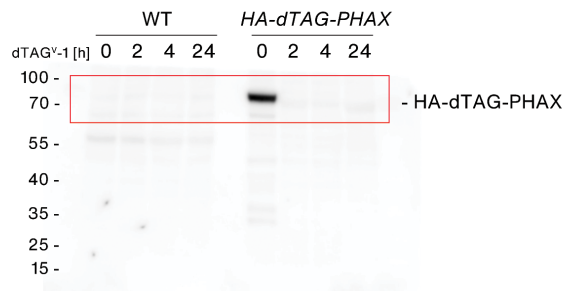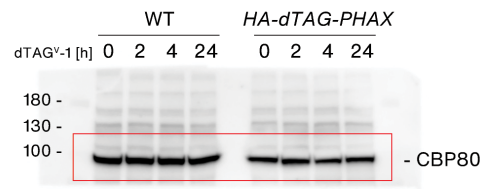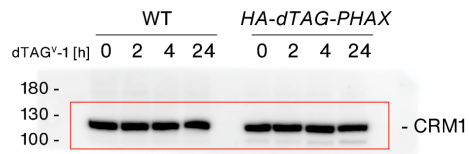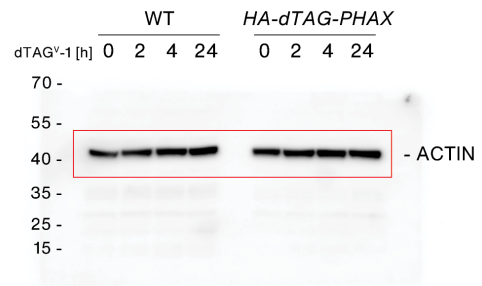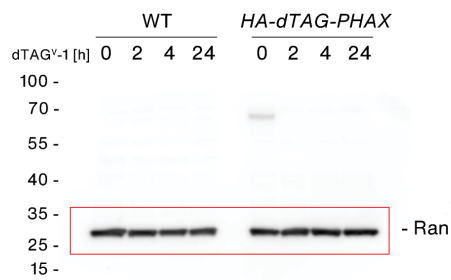

Extended data Figure 3e

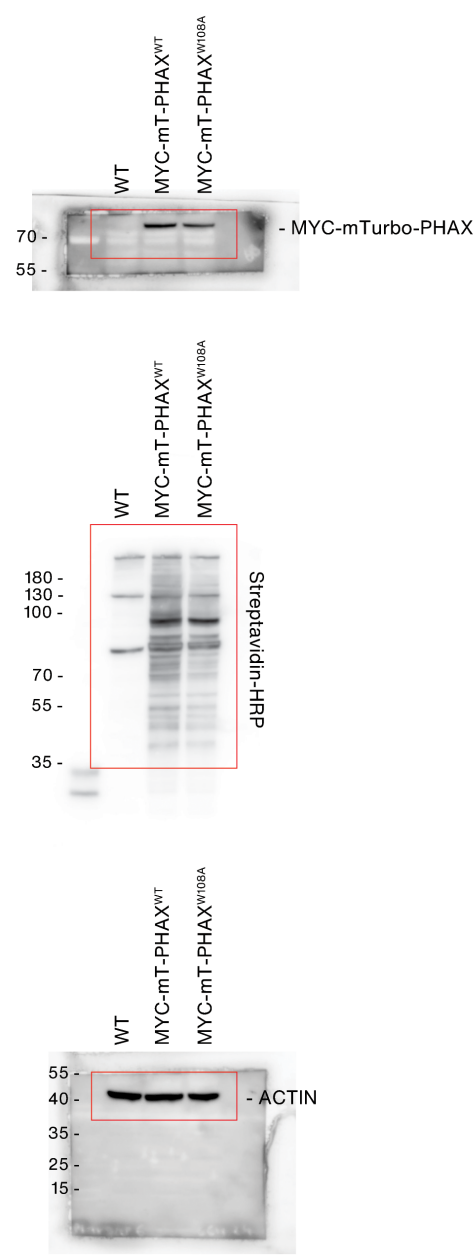

Supplement: Supplementary file 9 — Unprocessed western blots. [file 41594_2025_1595_MOESM9_ESM.pdf]

Extended data Figure 5a

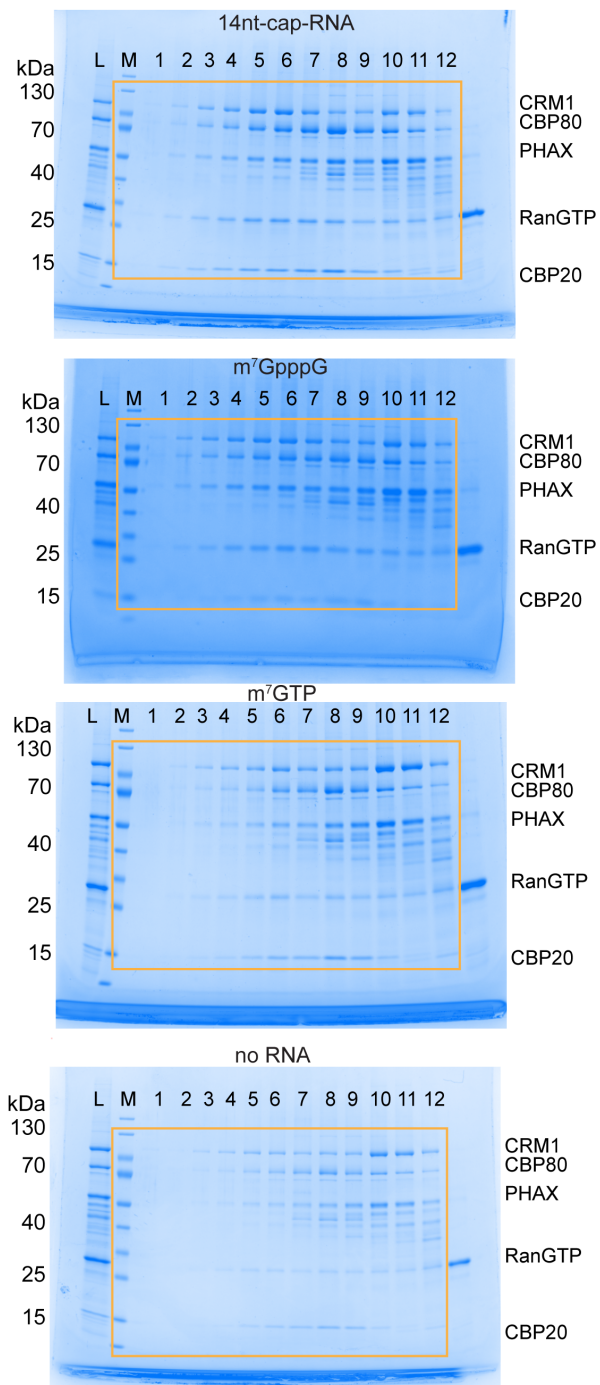

Extended data Figure 5e

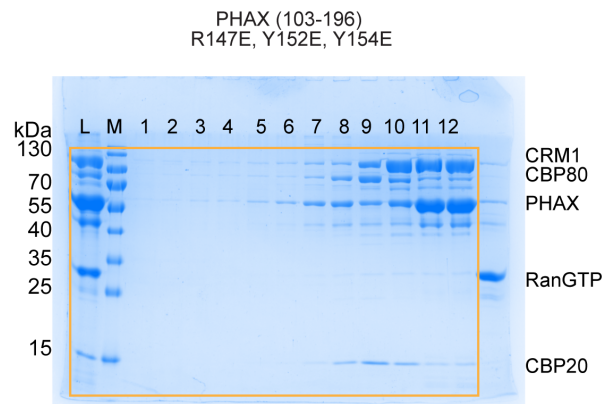

Supplement: Supplementary file 10 — Unprocessed gels. [file 41594_2025_1595_MOESM10_ESM.pdf]
